# SARS-CoV-2 genomic surveillance in Rondônia, Brazilian Western Amazon

**DOI:** 10.1038/s41598-021-83203-2

**Published:** 2021-02-12

**Authors:** Luan Felipo Botelho-Souza, Felipe Souza Nogueira-Lima, Tárcio Peixoto Roca, Felipe Gomes Naveca, Alcione de Oliveria dos Santos, Adriana Cristina Salvador Maia, Cicileia Correia da Silva, Aline Linhares Ferreira de Melo Mendonça, Celina Aparecida Bertoni Lugtenburg, Camila Flávia Gomes Azzi, Juliana Loca Furtado Fontes, Suelen Cavalcante, Rita de Cássia Pontello Rampazzo, Caio Henrique Nemeth Santos, Alice Paula Di Sabatino Guimarães, Fernando Rodrigues Máximo, Juan Miguel Villalobos-Salcedo, Deusilene Souza Vieira

**Affiliations:** 1 Oswaldo Cruz Foundation of Rondônia-FIOCRUZ/RO, Porto Velho, RO 76812 245 Brazil; 2 Rondônia Central Public Health Laboratory (LACEN/RO), Porto Velho, RO 76803-620 Brazil; 3 Leônidas and Maria Deane Institute (ILMD)-FIOCRUZ Amazonas, Manaus, AM 69027 070 Brazil; 4 Rondônia State Government, State Health Secretariat (SESAU/RO), Porto Velho, RO 76803-620 Brazil; 5 Institute of Molecular Biology of Paraná-IBMP, Curitiba, PR 81350-010 Brazil; 6 Tropical Medicine Research Center of Rondônia -CEPEM/RO, Porto Velho, RO 76812 329 Brazil; 7 Postgraduate Program in Experimental Biology, Federal University of Rondônia-PGBIOEXP/UNIR, Porto Velho, RO 76801 059 Brazil

**Keywords:** Computational biology and bioinformatics, Molecular biology

## Abstract

SARS-CoV-2 has spread rapidly around the world, with Brazil currently considered an epicenter of the pandemic. The Northern region has the second highest incidence coefficient, as well as the third highest mortality rate in the country. This study aimed to investigate information about the evolutionary history of epidemic spread and genetic aspects of strains isolated on the Western Amazon, in the State of Rondônia, Brazil. It was possible to detect a total of 22 mutations. Some of these alterations may possibly be related to effects on transmissibility, the fidelity of RNA replication, the ability of cancer patients to respond to infection, beyond a mutation that emerged after the introduction of SARS-CoV-2 in Rondônia. At least two events of introduction were detected, corresponding to the B.1 and B.1.1 European lineages. An introduction was observed possibly through Argentina, where strains originated that circulated in the Minas Gerais and Ceará Brazilian states, prior to Rondônia (B.1.), as well as through the Minas Gerais state and the Federal District, which gave rise to strains that spread to Rondônia, from the capital to more rural parts of the state (B.1.1.). The findings show the need to monitor the genetic epidemiology of COVID-19, in order to surveil the virus’s evolution, dispersion and diversity.

## Introduction

An outbreak of a serious respiratory disease of unknown etiology emerged in December 2019 in Wuhan, China. In early 2020, epidemiological and genetic analyses allowed for the identification of a new Coronavirus, later named Severe Acute Respiratory Syndrome Coronavirus 2 (SARS-CoV-2) which causes a clinical presentation now defined as Coronavirus Disease of 2019/COVID-19^1,2^. It is classified as part of the genus *Betacoronavirus*, subgenus *Sarbecovirus*, along with SARS-CoV, the specie responsible for the SARS outbreak in 2003^3^. Both have zoonotic origin associated with bats; the specific host responsible for the SARS-CoV-2 leap to the human species has not yet been determined, although there is evidence that the pangolin was the intermediate host^4,5^.

SARS-CoV-2 has a large and complex positive chain RNA genome of approximately 30,000 nucleotides in length that encodes known and hypothetical proteins^6^. Much of the viral genome corresponds to a large open reading frame (ORF), called ORF1ab. This gene encodes a polyprotein which, when cleaved during the replication process, gives rise to 16 non-structural proteins (nsp), including an RNA-dependent RNA polymerase (RdRp) and a helicase (Hel), as well as proteins involved in viral transcription, revision (Exonuclease nsp14), translation, cleavage (3CL-PRO), assembly and others related to the suppression of the host cell and immune system functions. The other part of the genome comprises genes encoding structural and accessory proteins, including the Spike surface glycoprotein (ORF2), made up of S1 and S2 domains, responsible for viral recognition and penetration through the human ACE-2 receptor; the membrane glycoprotein M (ORF5), responsible for the morphogenesis and assembly of the virus; nucleocapsid phosphoprotein N (ORF9), responsible for packaging genomic RNA, and envelope protein E (ORF4)^1,6–8^. Other reading frames are predictive and lack functional evidence. Thus, the viral genetic content has not been fully elucidated, with several predicted hypothetical open reading frames, whose function or even protein coding status is unknown^3^.

Since the outbreak began, SARS-CoV-2 has spread rapidly around the world. The virus has a high potential for transmissibility, with a reproductive number (R0) that can vary between 2.0 and 4.0^9^. Currently, four main transmission routes have been defined: (1) symptomatic transmission, which occurs through direct contact with a symptomatic individual; (2) environmental transmission, which occurs through sharing a contaminated environment or surface; (3) asymptomatic transmission, which occurs through direct contact with infected individuals who never presented symptoms and; (4) pre-symptomatic transmission, which occurs through direct contact with individuals who are in the incubation period (who do not yet have visible symptoms, a phase that can last on average 5 days, and may vary between 3 and 14 days). The latter is the main form of transmission, responsible for 42% to 66% of SARS-CoV-2 contamination, a rate sufficient to sustain an epidemic which, therefore, explains why social isolation restricted to symptomatic individuals is ineffective, and other strategies are necessary^10–14^.

Due to this ease and high rate of transmissibility, through January 07, 2021, COVID-19 has already been responsible for more than 1,890,000 deaths worldwide, with Brazil accounting for about 10.5% of this total (198,974)^15^, in addition to being considered by the World Health Organization (WHO) an epicenter of the pandemic in South America and in the world. The Central-West region of the country is considered the Brazilian epicenter, and currently presents the highest incidence and mortality rates of the country. However, the Northern region has the second highest incidence coefficient, as well as the third highest mortality rate (4562.7 and 96.1 per 100,000 inhabitants, respectively)^16^. Since the first confirmed case on March 20, 2020, Rondônia, a Brazilian state located in the southern part of the Amazon, has seen more than 98,000 confirmed cases (5209.6/100,000 inhabitants), and over 1875 deaths (98.1/100,000 inhabitants), with the great majority located in the capital, Porto Velho^16,17^. Although some Brazilian states have publicly disclosed genomic sequences of SARS-CoV-2, Rondônia does not yet have this type of information.

Extensive sequencing of the viral genome from different regions of the country provides insights into the prevalence of viral strains and any regional differences that may lead to a better understanding of patterns of transmission, outbreak tracking and, therefore, facilitate containment measures formulation. This study presents genetic data of the first 08 sequences of SARS-CoV-2 isolates in the state of Rondônia. The information available on the main mutations detected was reviewed in order to provide current and important details for the development of vaccines, specific antivirals and effective diagnostic tests. Additionally, the phylodynamic relationships between samples and sequences of isolates from different locations were also studied to assess the epidemic and evolutionary history of the virus in this State.

## Methods

### Nucleotide sequencing of viral isolates

The study was carried out by the Molecular Virology Laboratory of the Oswaldo Cruz Foundation of Rondônia (FIOCRUZ-RO) in collaboration with the Central Laboratory of Public Health of Rondônia (LACEN/RO) and the Leônidas and Maria Deane Institute (ILMD) of FIOCRUZ Amazonas. Ten samples of combined swabs collected from individuals residing in the State of Rondônia/Brazil with clinical symptoms of COVID-19 had detectable viral RNA following the RTq-PCR protocol from the Center of Disease Control and Prevention (CDC, Atlanta, USA). All samples were sequenced following the protocol described by Nascimento et al.^18^. In brief, samples were amplified with Platinum SuperFi II Green PCR master mix, visualized on agarose gel electrophoresis and precipitated with PEG 8000. All individual amplicons were quantified by fluorimetry and those belonging to the same sample were normalized and pooled. NGS libraries were constructed using the NexteraXT DNA and sequenced with MiSeq reagent kit v2 (500-cycles). Nucleotide sequencing was accomplished using the MiSeq (Illumina), installed at Fiocruz Amazônia, in a paired-end run (2 × 250 cycles). Raw data was converted to FASTQ using the Illumina pipeline at BaseSpace; trimmed for quality using BBDuk and finally assembled with BBMap embedded in Geneious v10.2.6, using the NC_045512 RefSeq as template. The number of mapped reads ranged between 297,808 and 1,093,088 with a mean coverage of 3,245X. Final sequences length ranged from 29,598 to 29,789 with 100% high-quality bases, as reported by Geneious statistics tool. Further details for each sequence, including Pangolin (https://pangolin.cog-uk.io); Nextclade (https://clades.nextstrain.org/) and CoVsurver reports (https://www.gisaid.org/epiflu-applications/covsurver-mutations-app/) are shown in the Supplementary Material (table S2).

### Ethics statement

Patients were informed in detail about the study and written consent was given by all participants. All clinical procedures and experiments were performed in accordance with international and national guidelines. The study was approved by the Research Ethics Committee of the Research Center for Tropical Medicine of Rondônia (CEP/CEPEM-RO), under opinion number 4000086.

### Analysis of mutations

Sequences of the viral isolates were aligned with the reference sequence for SARS-CoV-2 (NC_045512), a Wuhan isolate, in MEGA7 software (Molecular Evolutionary Genetics Analysis)^19^*,* under the action of the MUSCLE algorithm^20^. The positions where the nucleotide present in the samples diverged from that present in the reference sequence were marked as mutations.

### Determination of the evolutionary group

Some recent studies have identified large groups of strains of SARS-CoV-2. Forster et al.^21^, for example, using sequences available on the GISAID (Global Initiative on Sharing All Influenza Data) platform in early March, identified three groups of SARS-CoV-2 strains, named A, B and C. Another similar study, developed later by Yang et al.^22^, identified 4 large super-spreading groups (SS1-4), responsible for infections in most parts of the world. This latter classification was also used by Forster et al. (2020) and, therefore, this organization of super-spreading groups was used in the present study.

The previous classification of SARS-CoV-2 strains isolated in the state of Rondônia/Brazil in SS groups had two main objectives: (1) to stratify the amount of genetic data needed to carry out evolutionary analyses and (2) greater precision in the choice and use of evolutionary models and the molecular clock detailing the evolutionary history, permitting non-generalized performance in a more specific set of data. Therefore, 10 strains from each SS group were collected randomly and these, along with the 08 sample sequences, were included in a multiple sequence alignment performed by the online software MAFFT^23^ (https://mafft.cbrc.jp/alignment/software/). In order to increase the quality of phylogenetic inference, a root was also included in the alignment.

In this context, Pipes et al.^24^ published a recent study evaluating the uncertainty of rooting methods in the phylogeny of SARS-CoV-2. They concluded that neither the outgroup method using the bat CoV sequences RatG13 and RmYN02, nor the molecular clock method, presented reliable probabilities of root determination. However, through root-to-tip regression, a great posterior probability (0.516) of root placement in an old Wuhan sequence was observed (Wuhan / IPBCAMS-WH-01/2019—EPI_ISL_402123). Therefore, this sequence was also included in the alignment for rooting the inferred phylogeny. This alignment was called Dataset A and was made up of a total of 49 sequences.

Due to low homology at the extremities, the following analyses were performed based on the region of the genome that extends from position 55 to 29 838 (positions determined based on the reference sequence NC_045512). The alignment was used as an inference input for a non-clock tree based on Maximum Likelihood using the software IQtree v.1.6.12^25^. The replacement model was selected using the integrated Model Finder tool^26^ and based on the lowest Bayesian Information Criterion (BIC) value. The TIM + F + I model (transition model, with empirical frequency of bases and heterogeneity of invariant sites) was listed as most suitable for dataset A. Branch support values were obtained using 2000 replicas from Ultrafast Bootstrap^27^, with a search for nearest neighbor interchange (NNI) to optimize each initialization tree and reduce the risk of overestimating branch support. The generated consensus tree was imported into Tempest v.5.1 software^28^, in order to verify the existence of a temporal signal in this data set. Once the existence of a time signal is detected, the alignment then becomes sufficient for a molecular clock approach.

The molecular clock method was used for Bayesian inference of the phylogeny of evolutionary groups. The Lognormal and Exponential distributions of the Relaxed Non-Correlated Clock were tested. The replacement model used was the second best according to the estimate previously described, TN93 + F + I (Tamura-Nei with empirical base frequency and heterogeneity of invariant sites), since the TIM model is not among those supported in the BEAST v.1.10.4 package (SUCHARD et al., 2018). Phylogeny was calculated using the Coalescent: Exponential Growth model, through the Monte Carlo Markov Chain (MCMC), whose length was 1 × 10^7^ and with collections at every 1000, 10,000 samples were created. Convergence of the parameters was visualized in the software Tracer v.1.7^29^. The sample of trees was summarized and a consensus tree of maximum clade credibility (MCC) was built excluding 10% of the samples as burn-in using TreeAnnotator v.1.10.4 software. The consensus tree was visualized and customized using FigTree v.1.4.3.

### Detailing of the evolutionary history

Once the SS group of the strains was determined, the analyses continued to elucidate the detailed evolutionary history, through phylogenetic inference of the molecular clock with specific representatives of the group. Based on the results of the analysis of mutations and of the evolutionary group, it was observed that the study group would be SS4. Therefore, all SS4 representatives that were identified by Yang and collaborators (2020) were included in this second analysis.

Additionally, with exceptional help from the interactive panel of the GISAID platform, it was observed that the GISAID clades 20A, 20B and 20C comprise isolates that have the signature mutations of the SS4 group and, consequently, all sequences belonging to these clades were collected, using June 26th as the deadline. This collection was directed exclusively at strains isolated in South American countries, with Brazil being the only country whose sequences were collected at the State level. The use of this geographical filter at this stage of the study is justified due to the issue of urban mobility, a factor that may have been a facilitator and differential in the introduction of SARS-CoV-2 in the State of Rondônia.

Uncertainty and lack of resolution have been described regarding the phylogenies of SARS-CoV-2, due to the relatively small genetic diversity that has been accumulated during the short time of the outbreak^30^. Considering this, therefore, and in order to obtain good representativeness of genetic variability, locations with more than ten sequences available under the criteria previously mentioned, passed through two additional filters: (1) Maintain greatest disparity between the collection dates, to avoid sequences that are too similar and; (2) Maintain the representativeness of all pangolin lines available by location, ensuring a limited number of sequences per lineage, based on patient exposure location (States of Brazil: 10; other countries: 5). The whole process prioritized the highest degree of coverage between sequences when selecting them, not exceeding values less than 97.5% (considered average). At the end of the entire process, the alignment had a total of 307 sequences and was performed under the influence of the online software MAFFT. The region of analysis was the same as that previously used (nt 55—29 838). This second set of sequences was called Dataset B.

This phylogenetic inference followed a methodology similar to that used to determine evolutionary groups. In summary, a non-clock tree based on Maximum Likelihood was built using IQtree v.1.6.12 software under the previously described parameters. This, in turn, was used to visualize the existence of a temporal signal in the data set using the software Tempest v.1.5. Once a time signal was detected, the alignment was then imported into software from the BEAST package to perform a molecular clock approach. In this case, the Lognormal and Exponential distributions of the Non-Correlated Relaxed Clock were also tested. The replacement model used was GTR + F + I. The adjusted length of the MCMC for convergence of the parameters was 3 × 10^7^, with collection every 3000. The model used was Coalescent: Exponential Growth. The sample of trees was summarized, and the consensus tree was visualized/customized using FigTree v.1.4.4.

## Results and discussion

### Analysis of mutations

Of the ten samples from patients with a confirmed diagnosis for COVID-19, 8 were successful in the new generation sequencing procedure, generating complete genomic sequences with mean coverage level > 99%. The analysis of mutations in the genome of these strains demonstrated the presence of a total of 22 alterations in different sites, one of which was found in a non-coding region. Among those found in coding regions, 12 are classified as non-synonymous mutations. They are found in 7 viral proteins: nsp1, nsp12, Spike, ORF3a, ORF6, ORF8 and Nucleoprotein. The complete list of mutations found, and the percent frequency of occurrence is shown in Table 1. Percentage data on the frequency of mutations among the population were obtained from a comparison with isolates deposited in GISAID and collected on the timepoint of June 02, 2020.

**Table 1:** Mutations found in strains of SARS-CoV-2 isolated in Rondônia.

Mutation	Place of occurrence		Type of mutation	Proteic alteration	Freq. between study isolates	Freq. between population	
	Gene	Protein		(position at the gene level)		Brazil	Worldwide
**C241T**	5′ UTR	–	–	–	100%	100%	88%
C364A	ORF1ab	nsp1	Nonsynonymous	D33E	25%	0%	0%
**C3037T**	ORF1ab	nsp3	Synonymous	–	100%	100%	88%
C11563T	ORF1ab	nsp7	Synonymous	–	12.50%	0%	0%
C14265T	ORF1ab	nsp12 RdRp	Synonymous	–	12.50%	0%	0%
**C14408T**	ORF1ab	nsp12 RdRp	Nonsynonymous	P4715L	100%	100%	88%
C15324T	ORF1ab	nsp12 RdRp	Synonymous	–	25%	0%	5%
C16428T	ORF1ab	nsp13 Helicase	Synonymous	–	25%	0%	0%
T22156C	S	Spike/surface	Synonymous	–	12.50%	0%	0%
C23244A	S	Spike/surface	Nonsynonymous	P561H	25%	0%	0%
**A23403G**	S	Spike/surface	Nonsynonymous	D614G	100%	100%	87%
C23917T	S	Spike/surface	Synonymous	–	12.50%	0%	0%
T25036C	S	Spike/surface	Synonymous	–	12.50%	0%	0%
G25855T	ORF3a		Nonsynonymous	D155Y	25%	0%	0%
A26045G	ORF3a		Nonsynonymous	Q218R	25%	0%	0%
T27299C	ORF6		Nonsynonymous	I33T	75%	2%	4%
A28108C	ORF8		Nonsynonymous	Q72P	12.50%	0%	0%
G28881A	N	Nucleoprotein	Nonsynonymous	R203K	75%	100%	43%
G28882A	N	Nucleoprotein	Synonymous	-	75%	100%	43%
G28883C	N	Nucleoprotein	Nonsynonymous	G204R	75%	100%	43%
T29148C	N	Nucleoprotein	Nonsynonymous	I292T	75%	2%	4%
C29367T	N	Nucleoprotein	Nonsynonymous	P365L	37.50%	0%	0%

Four mutations were found in 100% of the isolates: C241T, C3037T, C14408T and A23408G. With the exception of alteration C14408T, the others were classified as signature mutations for super spreader group 4 (SS4) identified by Yang and collaborators (2020). Therefore, according to this information, all samples from the present study that were isolated belong to the SS4 group. Phylogenetic analysis to determine evolutionary groups (Fig. 2) confirms this classification.

The vast majority of the mutations found have no clinical/virological significance described in the literature, with some being considered unique. Among the known mutations in the ORF1ab gene, C14408T was found in 100% of the samples, which results in the replacement of a proline amino acid with a leucine at position 323 (P323L) of nsp12 RdRp (RNA-dependent RNA polymerase). Alterations in viral enzymes of this nature raise a level of concern, since they can cause resistance to drugs that have RdRp as a target, as previously described for hepatitis C, Influenza and also for one Coronavirus infection in mice treated with Rendesivir^31–33^.

However, the P323L alteration results in an amino acid with an isoelectric point similar to the wild type amino acid^34^, which may mean a not-so-significant change in the molecular structure of this protein. In addition, it is located outside the nsp12 RdRp catalytic region. However, it is located in a region equivalent to the SARS-CoV RdRp interface domain. This domain is supposedly implicated in the interaction with other viral proteins that can regulate the processivity of RdRp during the activity of Replicase Transcriptase Complex (RTC)^35,36^. RTC, in turn, has an interaction with the exonuclease nsp14, the protein responsible for reviewing viral RNA synthesis, and this interaction is important in the control of accurate RNA replication^35^. Thus, it is assumed that there is the possibility of an indirect influence of C14408T on the viral mutation rate.

In addition, it was recently proposed in a pre-printed study with over 11,200 sequences that this alteration may be associated with an increase in the rate of viral mutations^37^. In addition to this study, another also observed an extremely high rate of nucleotide substitutions in a group of viruses descending from an isolated parent strain in Germany (Germany/BavPat1/2020|EPI_ISL_406862), the SS4 group, which acquired the C14408T mutation later and was more widespread to other European countries^22^. Thus, considering the information raised about it and the high frequency of occurrence of this substitution in the analyzed samples, further studies are needed to assess the role of this mutation in the fidelity of RdRp, whose errors can directly affect the long-term effectiveness of a vaccine and specific antiviral drugs.

Among the modifications found in the S gene, A23403G stands out. This non-synonymous mutation was found in 100% of the samples and results in the replacement of an aspartate amino acid with a glycine at position 614 (D614G) of the Spike protein. This protein, through its receptor binding domain (RBD), mediates the interaction of the virus with the host cell by binding to ACE-2, which consequently facilitates membrane fusion and viral penetration^38,39^. The substitution results in an amino acid with an isoelectric point different from the wild type^34^, which may provide greater conformational freedom in the structure of the protein, improving local entropy and affecting the recognition interaction via RBD, through positioning of waste involved in this process^40^. This is one of the reasons this mutation has been a source of debate in the scientific literature regarding an association with possible higher transmissibility of SARS-CoV-2^34,40^.

Other reasons have also been pointed out to justify this association: (1) Structural reason: the resulting conformational change can improve the membrane fusion step by facilitating the separation of the S1 domain from the S2 domain of the Spike protein bound to the receptor; (2) Immune reason: because it is a residue located in a region equivalent to the target epitope of antibody-dependent improvement in SARS-CoV, it is assumed that the binding with the antibody may alter the conformation of the protein and increase its interaction with the ACE-2 receptor and; (3) Genetic epidemiological reason: an increase in the isolation frequency of strains that contain the D614G mutation has been detected in several regions of the world, including detection of the G614 variant's prevalence in a matter of weeks in places where the D614 variant was previously prevalent^34,41^.

Considering that this mutation was first identified on January 28, 2020 (Germany/BavPat1/2020|EPI_ISL_406862), this increase in frequency was also found in a direct genomic analysis of all 1539 SARS-CoV-2 genomes deposited in the GISAID platform between February 29th and March 26th. There was a prevalence of 56% of isolates belonging to the SS4 group, which hosts the D614G mutation as a signature characteristic of the group, showing the rapid dissemination of this variant over time^22^.

A recently published in vitro study that is currently in prepress performed comparisons of the functional properties of the D614 and G614 variations of the Spike protein, finding greater efficiency of infectivity with the G614 variant in the replication of pseudotyped retroviruses in cells that express ACE-2. The improvement was associated with a possible marked incorporation of Spike protein into the final structure of the virus, which may therefore improve the transmission of SARS-CoV-2 between different hosts^42^. Daniloski, Guo and Sanjana^43^ also came to the similar conclusion of a higher proportion of Spike protein per virion. Guo et al. (2020) showed that increased infectivity is associated with a change in Spike protein traffic towards lysosomes and away from organelles of the biosynthetic secretory pathway, in addition to inducing an increase in the level of processing of the S1-S2 junction during the replicative cycle^44^. In contrast, a pre-printed analysis of 15,691 SARS-CoV-2 genomes indicated that recurrent mutations in this protein did not increase viral transmissibility^45^.

A more current and comprehensive study brought new and relevant information to this discussion, by showing that the G614 variant has become dominant at the global level, in a way that suggests that such variant is undergoing positive selection. Additionally, it appears to be associated with a higher viral load in samples of the human upper respiratory tract and with greater infectivity in pseudotyping assays^46^. The interaction between A23403G and C14408T mutations may be related to greater viral fitness^47^. Both were found together in 100% of the analyzed samples, similar to that found by Liu et al. (2020) (74%)^48^, that suggested there is a biological connection between these changes.

Clinically, D614G does not appear to be associated with the severity of the disease^41^. This can be justified by the fact that the G614 variation provides limitations to the rate and efficiency of intra-host replication^42^. Although the number of samples in the present study may be considered low, none of the individuals with the strains analyzed died of the infection and only one (12.5%) developed a clinical condition considered severe (dyspnoic). This percentage rate is similar to the general epidemiological rate of clinical resolution of COVID-19, showing no link between D614G and the severity of the disease.

Two changes in different genes were found together in 75% of the samples: T27299C and T29148C. Both are classified as non-synonymous mutations that result in the substitution of an isoleucine amino acid with a threonine at positions 33 (I33T) and 292 (I292T) of the viral proteins ORF6 and N, respectively. According to the study by Candido et al.^49^, the coexistence of these two mutations corresponds to the signature of one of three main clades of SARS-CoV-2 spread in Brazil, named clade 2. This clade is the most spatially disseminated strain in the country, with isolated representatives in a total of 16 of the 26 Brazilian states as of the end of April 2020. However, this same study did not obtain a good representation of the northern region of the country, with the absence of genetic data of circulating strains in Rondônia. Therefore, we have detected the spread of this clade to yet another Brazilian state. Phylogenetic analyses developed in the present study that will be presented and discussed later showed that this group of samples corresponds to pangolin line B.1.1., although clade 2 identified by Candido et al.^49^ may include other pangolin lineages. The authors did not specify the pangolin lines included in each of the three main clades detected.

Other changes were also found in the N gene of the strains analyzed. Three sequential nucleotide changes are highlighted: G28881A, G28882A and G28883C, which were found together in 75% of the samples. They result in the replacement of two amino acids of the viral Nucleoprotein, R203K and G204R (R—arginine; K—lysine; G—glycine). The potential effect of these mutations on viral and host processes has been investigated, and it has been observed that they result in considerable changes in the predicted binding with some miRNAs, which may play a role in influencing the progress of the infection. Some of the miRNAs that bind to this mutated type of nucleoprotein may be under-regulated in several types of cancer. This increases the possibility that cancer patients may have a high susceptibility to the mutated variant due to a reduced ability to contain the virus, compared to the wild-type infection^40^.

Another alteration detected in the same gene is C29367T, found in three (37.5%) of the eight samples in the study. It is a nonsynonymous mutation that results in a P365L substitution. This mutation has not yet been described in the scientific literature. When looking for other sequences with this mutation in Dataset B, it was observed that none of the strains included show this change. This leads to the assumption that it has appeared more recently in the viral evolutionary history of SARS-CoV-2. Because it was detected only in some sequences in Rondônia, it may have appeared after the virus entered the state and can be used as a marker to study viral spread among different municipalities in the state.

### Evolutionary analysis

#### Temporal signal evaluation

**Figure 1:**
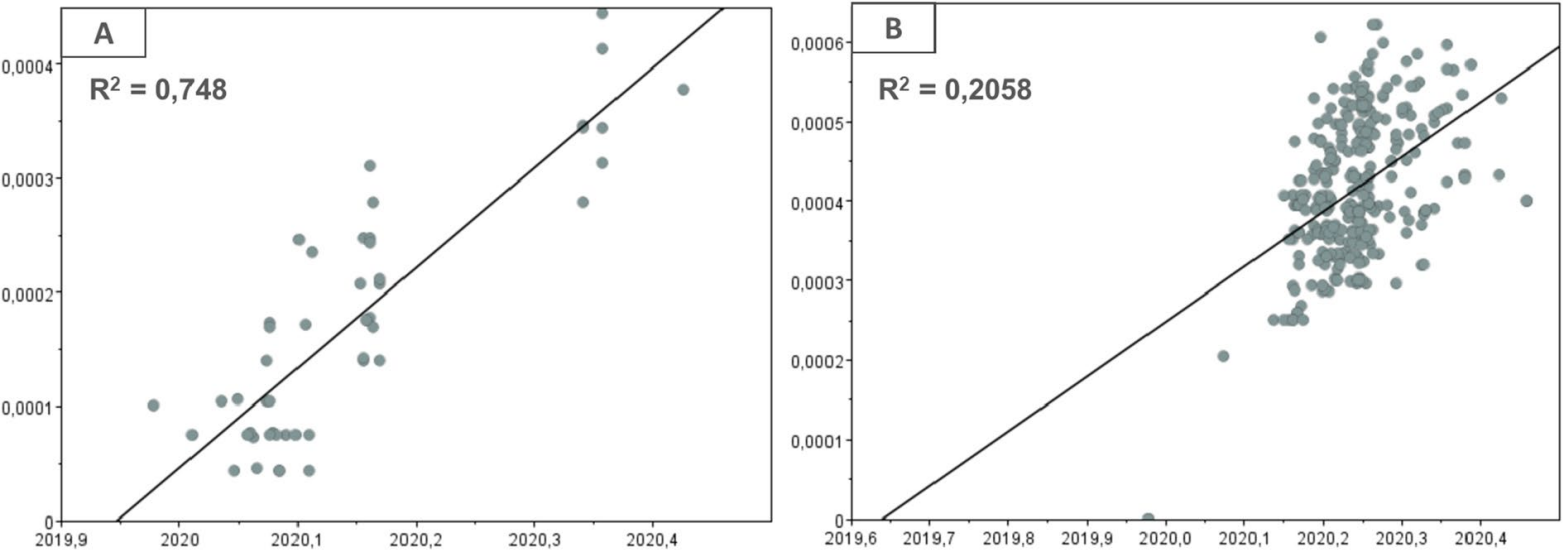
Linear regression graphs of temporal signal detection. The graphs show the positive correlation between genetic diversity from root to tip (y-axis) and the sampling time of the included sequences (x-axis). This effect on the relationship of these variables shows the existence of a temporal signal in the analyzed data set, which makes it sufficient for molecular clock analysis. Graphs A and B refer to the analyses in datasets A and B, respectively. The value of R^2^ is shown in the upper left corner of the corresponding graph.

For both Dataset A and Dataset B, it was possible to observe a linear regression curve that shows a positive correlation between genetic diversity and sampling time, showing the existence of sufficient time signal in the data sets to justify a molecular clock approach (Fig. 1, A and B). Although the time signal level may be considered low for Dataset B, as evidenced by the R^2^ value (0.2058), this parameter should not be used to test the statistical significance of the regression, because the individual data points in the graph are not distributed independently, but are partially correlated due to shared phylogenetic ancestry^28^.

#### Determination of evolutionary group

**Figure 2:**
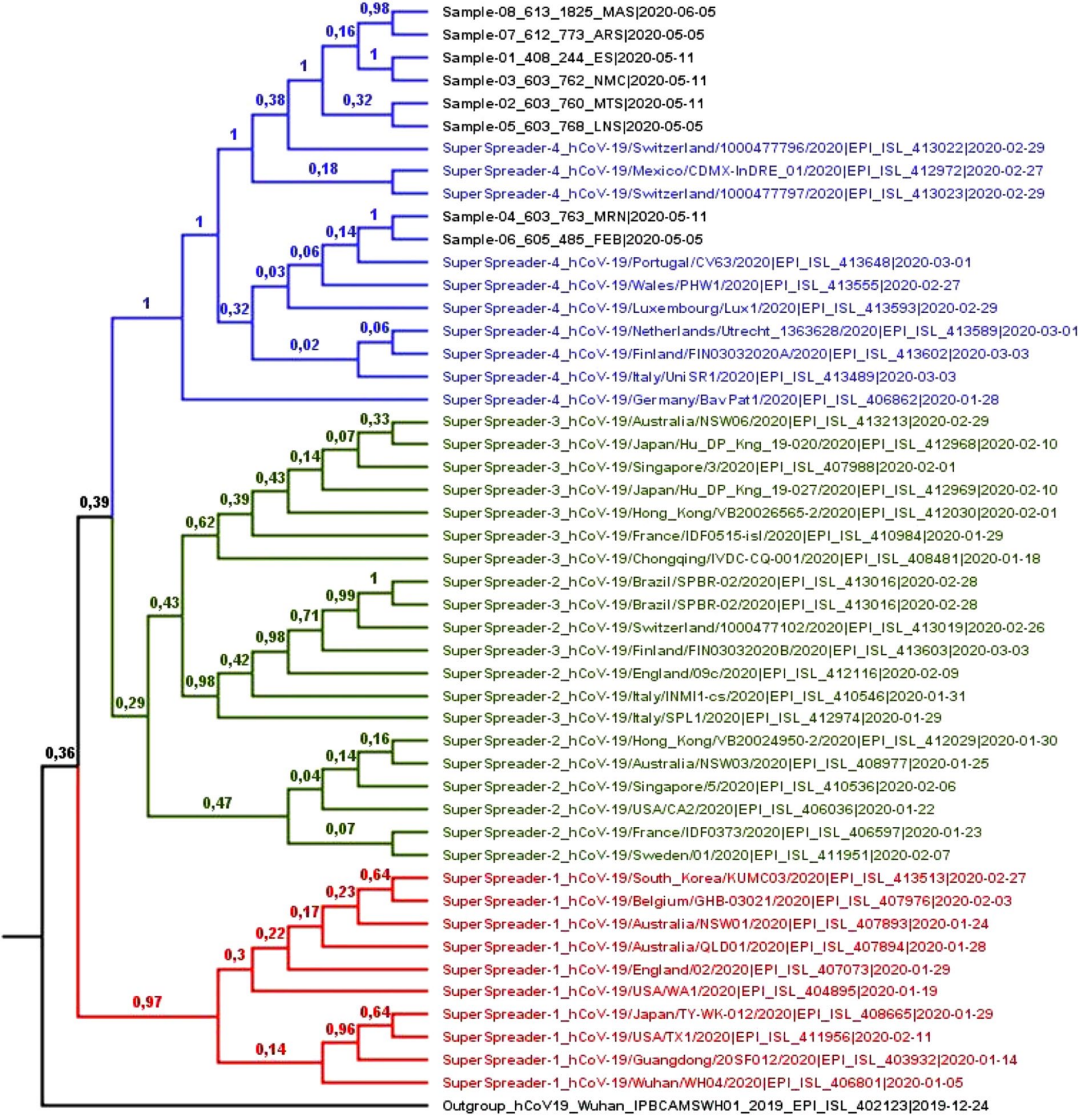
Bayesian phylogenetic analysis to determine evolutionary group. In the generated MCC tree, the phylogenetic relationship was estimated from 49 SARS-CoV-2 sequences included in dataset A. The red taxa correspond to the SS1 group; groups SS2 and SS3 are in green, mixed as previously proposed and; the SS4 group is in blue. The study samples are colored black, as well as the sequence used for rooting the inferred tree. In each node, the subsequent probability rate for supporting the branches in decimal data is shown. The time for the most recent common ancestor (tMRCA) among all variations of SARS-CoV-2 was dated to October 23, 2019 (95% Highest Posterior Density—HPD: July 29 and December 17, 2019), similar to other studies^49–51^, which shows the accuracy of the molecular clock addressed in the present study.

Phylogenetic analysis to determine evolutionary groups of SARS-CoV-2 strains in the present study, using dataset A, confirmed one of the conclusions previously obtained with the study of mutations: all samples were identified as belonging to the SS4 group, with a posterior support of the 100% cladistic distribution (Fig. 2). The best distribution of the non-correlated relaxed clock was “exponential”, chosen through the analysis of convergence of MCMC run parameters and tree topology.

According to the evolutionary history of the SS groups pointed out by Yang et al. (2020), strains descended from the original virus were transmitted to various locations in the world and were dominant for a period of time, during the early outbreak of COVID-19. However, with continuous transmission in different environments, the virus has evolved into four large super-spreading clusters, along with other variants derived directly from the original virus. SS group members became dominant, with different variants prevailing in different regions of the world, in mid-February and March.

The SS1 strains first emerged and were transmitted mainly in Asia, South Korea and the USA. They persisted in China during the post-initial outbreak phase, being less prevalent in other parts of the world. Groups SS2 and SS3 were transmitted mainly in mid-January and February, in Asian countries other than China, as well as Europe and Brazil, specifically in the State of São Paulo during the initial phase of the outbreak. Finally, group SS4 emerged in late January and was reported for the first time in Germany. It was primarily responsible for the outbreak of a pandemic on the European continent, replacing the previous dominance of strains SS2 and SS3 in the region. From this continent, this variant has spread to several other locations around the world, as already discussed in relation to the D614G substitution. It also arrived in South America where, in mid-March, it entered the State of Rondônia.

This analysis allowed us to observe that at least two different events of entry occurred in the State, both of European descent. It also showed a deficiency of phylogenetic signal to differentiate strains from groups SS2 and SS3. In fact, for identification through direct genomic observation, both groups have only one signature mutation each (G26144T for SS2 and G11083T for SS3), which may show little phylogenetically useful difference for differentiating strains from these groups, when considering the integral size of the SARS-CoV-2 genome and its biological tendency to maintain conservation. In addition, we observed some Brazilian strains deposited in Genbank (MT126808.1 and MT350282.1) that have both of the aforementioned substitutions. Therefore, we suggest the union of groups SS2 and SS3 in the classification of super spreaders. Fortunately, this question does not negatively influence the determination of the samples as descendants of the SS4 group.

#### Detailed evolutionary history

**Figure 3:**
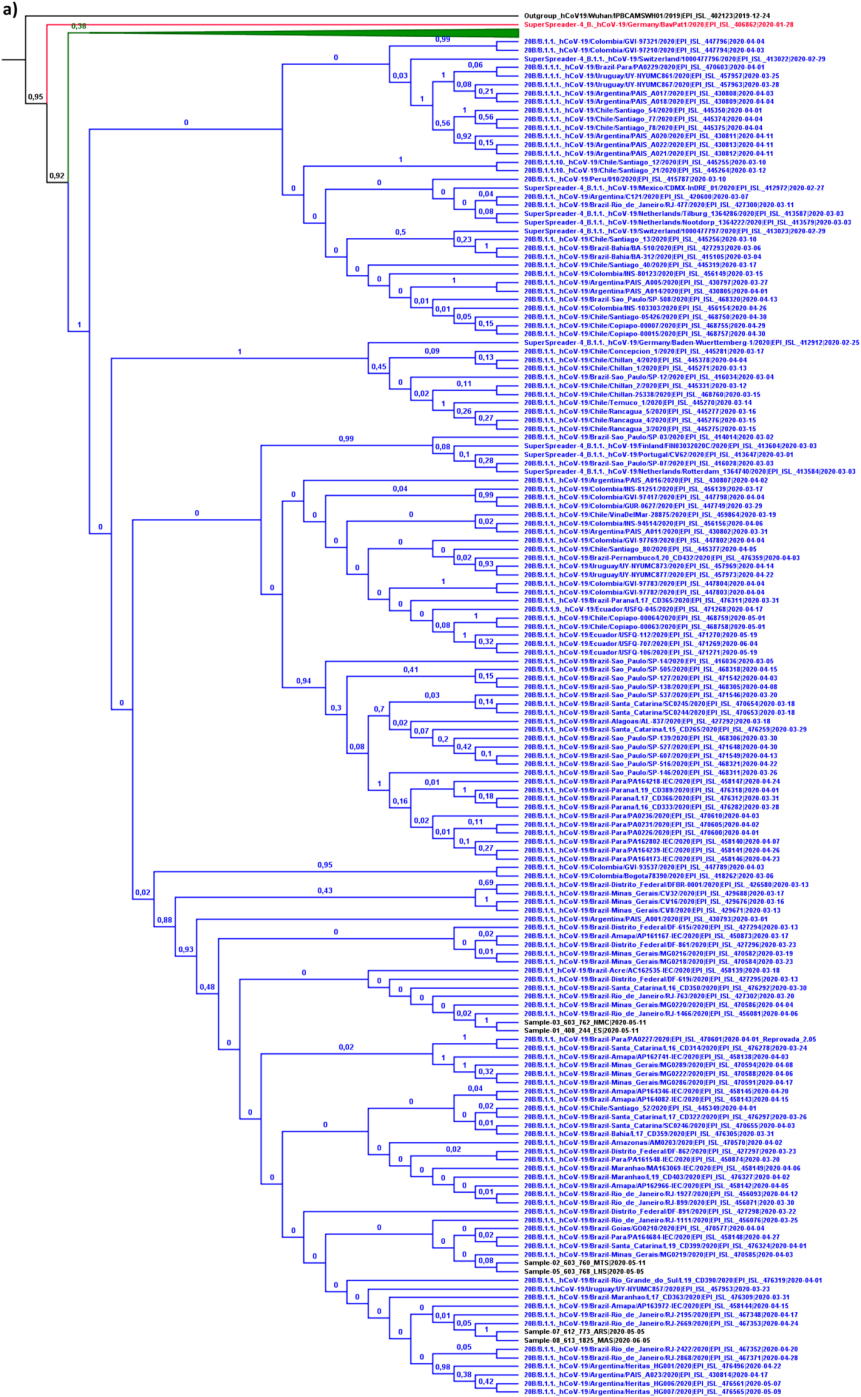

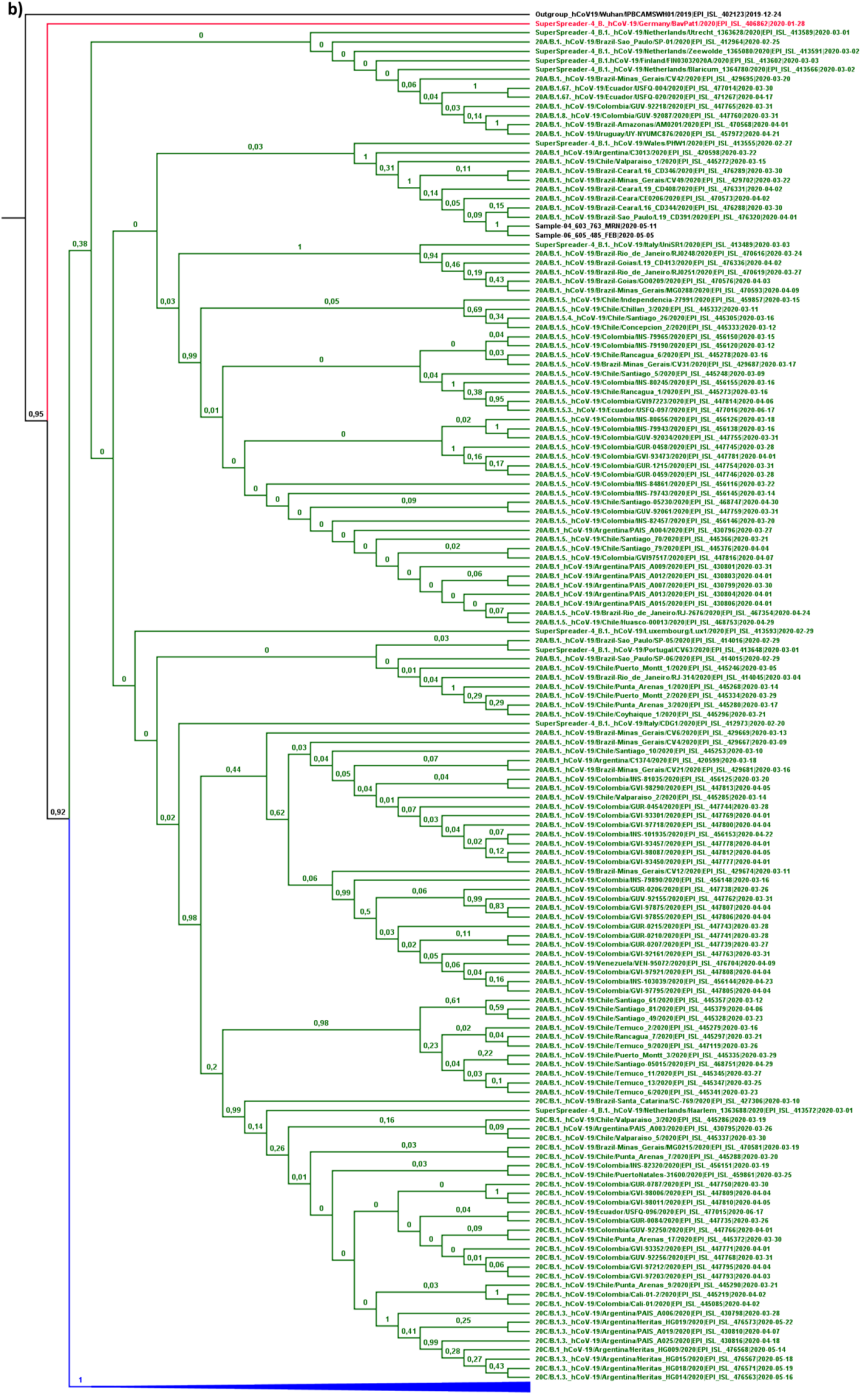
Bayesian phylogenetic tree for detailing the evolutionary path. In the generated MCC tree, the phylogenetic relationship was estimated based on 307 SARS-CoV-2 sequences included in dataset B. (**a**) The taxa and clades colored in blue correspond to strains belonging to variations of pangolin lineage B.1.1., including: B.1.1., B.1.1.1., B.1.1.10. and B.1.1.9. (**b**) The green colored taxa and clades correspond to strains belonging to other variations of pangolin lineage B.1., Including: B.1., B.1.3., B.1.5., B.1.5.4., B. 1.67. and B.1.8. The study samples are colored black, along with the sequence used for rooting the inferred tree. In each node, the subsequent probability rate for supporting the branches in decimal data is shown. The tMRCA among all variations of SARS-CoV-2 was dated to November 20, 2019 (95% HPD between October 17 and December 20, 2019), similar to other studies^49–51^, which may show the accuracy of the molecular clock addressed in the present study.

Phylogenetic analysis to detail the evolutionary history of the SARS-CoV-2 strains from the present study was performed based on the relaxed correlated molecular clock model using dataset B. The “lognormal” distribution was chosen through the convergence analysis of MCMC run parameters and tree topology. The inferred tree allowed us to observe that 75% of the strains isolated in the State of Rondônia belong to pangolin lineage B.1.1.; while the remaining 25% belong to line B.1. (Fig. 3A,B). This classification was supported by 100% of subsequent probability in determining the lineage at some cladistic level of the tree and provides support for the previous conclusion of the occurrence of at least two SARS-CoV-2 entry events in the State.

In order to avoid inaccurate conclusions regarding the introduction of SARS-CoV-2 in the State, details of the evolutionary path were obtained from information on clades that included samples with posterior support greater than 85%. Therefore, considering this criterion, it was not possible to fully detail the evolutionary history of the introduction of the B.1.1 strain. In addition to being of European descent, B.1.1. strains from the state of Rondônia also descend from an ancestral strain that circulated in Argentina around the transition from February to March, with a differentiation date of February 25th (95% HPD between February 14th and 29th (Fig. 3A). It was not possible to draw any further conclusions about the detailed path between the transmission from Argentina to Rondônia, nor whether it occurred directly between these localities.

However, another interpretation is also possible. This group of sequences share a common ancestor, descended from an older one (dated February 15th, with 95% HPD between January 28th and February 26th) that gave rise to isolated strains in the middle of March in the state of Minas Gerais and the Federal District. Therefore, it is possible that strains circulating in these states have spread to Argentina and Rondônia. A previous study identified the transmission of B.1.1. strains to some South American countries, including Argentina^52^. This supports the second hypothesis surrounding the introduction of this lineage into the State.

The detailing of the evolutionary path regarding the introduction of the B.1. line provided more detailed information about this process. Just like for B.1.1., B.1. strains also share ancestry with a parental strain that circulated in Argentina, having differentiated from a common ancestor on February 29th (95% HPD between February 26th and March 15th). Another more recently shared common ancestor gave rise to strains that circulated in the Brazilian states of Minas Gerais and Ceará, dated March 9th (95% HPD between March 8th and 21st) (Fig. 3B). This last dating does not represent the exact period of arrival of this lineage in the State, but a period close to this event. It should be noted that the first confirmed case in the state of Rondônia occurred on March 20th.

Three pairs of samples are lined up in the analysis in a monophyletic manner with 100% posterior support, showing a very high degree of similarity between them. This shows the expected effect of sustained community transmission of the virus in the state. The monophyletic relationship of B.1.1. strains of the sample pairs 01–03 and 07–08 may provide relevant information about the viral dissemination profile in the State. With the exception of sample 07, the others have the aforementioned C29367T alteration in their genome, which presumably arose after the introduction of SARS-CoV-2 in the State and which can be used as a source of information to study the form of dissemination. Therefore, it is presumed that this alteration occurred after passing, not necessarily directly, from 07 to 08 in the city of Porto Velho (place of residence of their respective carriers). Subsequently, there was a continuation of the transmission of strains that carry this mutation before reaching the list of samples 01–03. Since sample 03 was isolated from a patient residing in Porto Velho, and sample 01 was isolated from a patient residing in the municipality of Jaru (about 290 km away from the capital), it is assumed that a strain was transmitted from Porto Velho to Jaru.

## Conclusion

This study presented the genetic data of the first 08 SARS-CoV-2 sequences isolated in the state of Rondônia/Brazil, located in the southern portion of the Western Amazon. It was possible to determine at least two events of viral introduction into the state, corresponding to strains B.1. and B.1.1., around the transition from February to March 2020. In addition to both strains being of European descent, another possible introduction was observed through Argentina, passing through the Brazilian states of Minas Gerais and Ceará (B.1.), as well as from Minas Gerais and the Federal District to Argentina and Rondônia (B.1.1.).

Despite limitations resulting from the low number of samples analyzed in this study, genetic mapping allowed us to observe the presence of a total of 22 mutations. Some of these changes may possibly be related to higher transmissibility effects (A23403G/D614G/Spike glycoprotein), influence RNA replication fidelity (C14408T/P323L/nsp12 RdRp), influence the ability of cancer patients to respond to infection (G28881A, G28882A and G28883C/R203K and G204R/Nucleoprotein), in addition to a mutation (C29367T, P365L, Nucleoprotein) that emerged after the introduction of SARS-CoV-2 in the state of Rondônia, which may represent adaptation to environmental and human conditions. This information is important because it provides current and essential details for the development of vaccines, specific antivirals and effective diagnostic tests.

The findings highlight the importance of implementing a surveillance system for the genetic epidemiology of the virus in the State, which may permit the monitoring of viral evolution and dissemination in the capital and in other regions of the State through obtaining more genome sequences of the circulating strains. This can provide insights into the prevalence of viral strains and regional differences in patterns of transmission, epidemiological screening and formulation of containment measures.

## Supplementary Information

Supplementary Legends.

Supplementary Table S1.

Supplementary Table S2.

## Data Availability

The datasets generated during the current study are available in the GISAID (Global Initiative on Sharing All Influenza Data) platform repository, under the access numbers EPI_ISL_514131 to EPI_ISL_514138. The informations about collected sequences used in this study are available on the Supplementary Material (table S1). The other data generated during the development of the study are available together in a public repository (https://doi.org/10.17632/dnh8jpz6cn.1), containing the necessary files for analyzes, specifically the alignments used, as well as the results files generated at each stage of the research.
