# Supplementary material for: SARS-CoV-2 genomic surveillance in Rondônia, Brazilian Western Amazon
Source: Sci Rep. 2021 Feb 12;11:3770. doi: 10.1038/s41598-021-83203-2 (PMC7881028; doi:10.1038/s41598-021-83203-2)
Supplement: Supplementary file 1 — Supplementary Legends. [file 41598_2021_83203_MOESM1_ESM.docx]

# ****Supplemental files to:****

# ****SARS-Cov-2 genomic surveillance in Rondônia, Brazilian Western Amazon****

**Luan Felipo Botelho-Souza^1,2,#,*^, Felipe Souza Nogueira-Lima^1,7,#^, Tárcio Peixoto Roca^1,7,#^, Felipe Gomes Naveca^3^, Alcione de Oliveira dos Santos^1,2^ , Adriana Cristina Salvador Maia^4^, Cicileia Correia da Silva^4^, Aline Linhares Ferreira de Melo Mendonça^4^, Celina Aparecida Bertoni Lugtenburg^2,4^, Camila Flávia Gomes Azzi^4^, Juliana Loca Furtado^2,4^, Suelen Cavalcante^2,4^,**

**^1^** Oswaldo Cruz Foundation of Rondônia - FIOCRUZ/RO, Porto Velho RO 76812 245, Brazil;

**^2^** Rondônia Central Public Health Laboratory (LACEN / RO), Porto Velho RO, 76803-620, Brazil;

**^3^** Leônidas and Maria Deane Institute (ILMD) - FIOCRUZ Amazonas, Manaus AM 69027 070, Brazil;

**^4^** Rondônia State Government, State Health Secretariat (SESAU / RO), Porto Velho RO, 76803-620, Brazil;

**^5^** Institute of Molecular Biology of Paraná -IBMP, Curitiba 81350-010, PR, Brazil;

**^6^** Tropical Medicine of Rondônia Research Center - CEPEM/RO, Porto Velho RO 76812 329, Brazil;

^7^ Posgraduate Program in Experimental Biology of Federal University of Rondônia-PGBIOEXP, Porto Velho RO 76801 059, Brazil

# Contributed equally

* Correspondence should be addressed: deusilene.vieira@fiocruz.br

**This zip contains two files corresponding to:**

**Table S1:** The acknowledgments to GISAID platform and collaborators

**Table S2:** Sequencing data
