# Supplementary material for: SARS-CoV-2 genomic surveillance in Rondônia, Brazilian Western Amazon
Source: Sci Rep. 2021 Feb 12;11:3770. doi: 10.1038/s41598-021-83203-2 (PMC7881028; doi:10.1038/s41598-021-83203-2)
Supplement: Supplementary file 2 — Supplementary Table S1. [file 41598_2021_83203_MOESM2_ESM.pdf]

We gratefully acknowledge the following Authors from the Originating laboratories responsible for obtaining the specimens and the Submitting laboratories where genetic sequence data were generated and shared via the GISAID Initiative, on which this research is based.

Virus name	Accesion number	Collected	Originating Lab	Submitting Lab	Author
**Wuhan/IPBCAMS-WH-01/2019**	EPI_ISL_402123	2019-12-24	Institute of Pathogen Biology, Chinese Academy of Medical Sciences & Peking Union Medical College	Institute of Pathogen Biology, Chinese Academy of Medical Sciences & Peking Union Medical College	Ren et al.
**Australia/NSW01/2020**	EPI_ISL_407893	2020-01-24	Centre for Infectious Diseases and Microbiology Laboratory Services	NSW Health Pathology - Institute of Clinical Pathology and Medical Research; Westmead Hospital; University of Sydney	Eden et al.
**Guangdong/20SF012/2020**	EPI_ISL_403932	2020-01-14	Guangdong Provincial Center for Diseases Control and Prevention; Guangdong Provincial Public Health	Department of Microbiology, Guangdong Provincial Center for Diseases Control and Prevention	Kang et al.
**Japan/TY-WK-012/2020**	EPI_ISL_408665	2020-01-29	Dept. of Virology III, National Institute of Infectious Diseases	Pathogen Genomics Center, National Institute of Infectious Diseases	Sekizuka et al.
**USA/TX1/2020**	EPI_ISL_411956	2020-02-11	Texas Department of State Health Services	Pathogen Discovery, Respiratory Viruses Branch, Division of Viral Diseases, Centers for Dieases Control and Prevention	Queen et al.
**USA/WA1/2020**	EPI_ISL_404895	2020-01-19	Providence Regional Medical Center	Division of Viral Diseases, Centers for Disease Control and Prevention	Queen et al.
**Belgium/GHB-03021/2020**	EPI_ISL_407976	2020-02-03	KU Leuven, Clinical and Epidemiological Virology	KU Leuven, Clinical and Epidemiological Virology	Vanmechelen et al.

**South Korea/KUMC03/2020**	EPI_ISL_413513	2020-02-27	Division of Infectious Diseases, Department of Internal Medicine, Korea University College of Medicine	Department of Microbiology, Institute for Viral Diseases, College of Medicine, Korea University	Kang et al.
**England/02/2020**	EPI_ISL_407073	2020-01-29	Respiratory Virus Unit, Microbiology Services Colindale, Public Health England	Respiratory Virus Unit, Microbiology Services Colindale, Public Health England	Galiano et al.
**Australia/QLD01/2020**	EPI_ISL_407894	2020-01-28	Pathology Queensland	Public Health Virology Laboratory	Huang et al.
**Wuhan/WH04/2020**	EPI_ISL_406801	2020-01-05	General Hospital of Central Theater Command of People's Liberation Army of China	BGI & Institute of Microbiology, Chinese Academy of Sciences & Shandong First Medical University & Shandong Academy of Medical Sciences & General Hospital of Central Theater Command of People's Liberation Army of China	Chen et al.
**Australia/NSW03/2020**	EPI_ISL_408977	2020-01-25	Serology, Virology and OTDS Laboratories (SAViD), NSW Health Pathology Randwick	NSW Health Pathology - Institute of Clinical Pathology and Medical Research; Centre for Infectious Diseases and Microbiology Laboratory Services; Westmead Hospital; University of Sydney	Eden et al.
**Brazil/SPBR-02/2020**	EPI_ISL_413016	2020-02-28	Hospital Israelita Albert Einstein	Instituto Adolfo Lutz, Interdisciplinary Procedures Center, Strategic Laboratory	Jesus et al.
**England/09c/2020**	EPI_ISL_412116	2020-02-09	Respiratory Virus Unit, Microbiology Services Colindale, Public Health England	Respiratory Virus Unit, Microbiology Services Colindale, Public Health England	Galiano et al.

**France/IDF0373/2020**	EPI_ISL_406597	2020-01-23	Department of Infectious and Tropical Diseases, Bichat Claude Bernard Hospital, Paris	National Reference Center for Viruses of Respiratory Infections, Institut Pasteur, Paris	Albert et al.
**Hong_Kong/VB20024950-2/2020**	EPI_ISL_412029	2020-01-30	Hong Kong Department of Health	The University of Hong Kong	Dominic et al.
**Italy/INMI1-cs/2020**	EPI_ISL_410546	2020-01-31	INMI Lazzaro Spallanzani IRCCS	Laboratory of Virology, INMI Lazzaro Spallanzani IRCCS	Capobianchi et al.
**Singapore/5/2020**	EPI_ISL_410536	2020-02-06	Singapore General Hospital, Molecular Laboratory, Division of Pathology	Programme in Emerging Infectious Diseases, Duke-NUS Medical School	Anderson et al.
**Sweden/01/2020**	EPI_ISL_411951	2020-02-07	unknown	Unit for Laboratory Development and Technology Transfer, Public Health Agency of Sweden	Bengner et al.
**Switzerland/1000477102/2020**	EPI_ISL_413019	2020-02-26	Department of Internal Medicine, Triemli Hospital	Institute of Medical Virology, University of Zurich	Schmutz et al.
**USA/CA2/2020**	EPI_ISL_406036	2020-01-22	California Department of Public Health	Pathogen Discovery, Respiratory Viruses Branch, Division of Viral Diseases, Centers for Dieases Control and Prevention	Uehara et al.
**Australia/NSW06/2020**	EPI_ISL_413213	2020-02-29	Centre for Infectious Diseases and Microbiology Laboratory Services	NSW Health Pathology - Institute of Clinical Pathology and Medical Research; Westmead Hospital; University of Sydney	Eden et al.
**Brazil/SPBR-02/2020**	EPI_ISL_413016	2020-02-28	Hospital Israelita Albert Einstein	Instituto Adolfo Lutz, Interdisciplinary Procedures Center, Strategic Laboratory	Jesus et al.
**Chongqing/IVDC-CQ-001/2020**	EPI_ISL_408481	2020-01-18	National Institute for Viral Disease Control and Prevention, China CDC	National Institute for Viral Disease Control & Prevention, CCDC	Tan et al.
**Finland/FIN03032020B/2020**	EPI_ISL_413603	2020-03-03	Department of Virology and Immunology, University of	Department of Virology, Faculty of Medicine,	Smura et al.

			Helsinki and Helsinki University Hospital, Huslab Finland	University of Helsinki, Helsinki, Finland	
**France/IDF0515-isl/2020**	EPI_ISL_410984	2020-01-29	Department of Infectious and Tropical Diseases, Bichat Claude Bernard Hospital, Paris	National Reference Center for Viruses of Respiratory Infections, Institut Pasteur, Paris	Albert et al.
**Hong Kong/VB20026565-2/2020**	EPI_ISL_412030	2020-02-01	Hong Kong Department of Health	School of Public Health, The University of Hon g Kong	Dominic et al.
**Italy/SPL1/2020**	EPI_ISL_412974	2020-01-29	Department of Infectious Diseases, Istituto Superiore di Sanità, Rome, Italy	Virology Laboratory, Scientific Department, Army Medical Center	Stefanelli et al.
**Japan/Hu_DP_Kng_19-020/2020**	EPI_ISL_412968	2020-02-10	unknown	Takayuki Hishiki Kanagawa Prefectural Institute of Public Health, Department of Microbiology	Hishiki et al.
**Japan/Hu_DP_Kng_19-027/2020**	EPI_ISL_412969	2020-02-10	unknown	Takayuki Hishiki Kanagawa Prefectural Institute of Public Health, Department of Microbiology	Hishiki et al.
**Singapore/3/2020**	EPI_ISL_407988	2020-02-01	National Centre for Infectious Diseases	Programme in Emerging Infectious Diseases, Duke-NUS Medical School	Anderson et al.
**Finland/FIN03032020A/2020**	EPI_ISL_413602	2020-03-03	Department of Virology and Immunology, University of Helsinki and Helsinki University Hospital, Huslab Finland	Department of Virology, Faculty of Medicine, University of Helsinki, Helsinki, Finland	Teemu Smura, Hannimari Kallio-Kokko, Olli Vapalaht
**Switzerland/1000477797/2020**	EPI_ISL_413023	2020-02-29	Division of Infectious Diseases, University Hospital Zurich	Institute of Medical Virology, University of Zurich	Schmutz et al.
**Switzerland/1000477796/2020**	EPI_ISL_413022	2020-02-29	Division of Infectious Diseases, University Hospital Zurich	Institute of Medical Virology, University of Zurich	Schmutz et al.
**Portugal/CV62/2020**	EPI_ISL_413647	2020-03-01	Centro Hospital do Porto, E.P.E. - H. Geral de Santo Antonio	Instituto Nacional de Saude (INSA)	Guiomar et al.

**Netherlands/Tilburg_1364286/2020**	EPI_ISL_413587	2020-03-03	Foundation Elisabeth-Tweesteden Ziekenhuis	Erasmus Medical Center	Nieuwenhuijse et al.
**Netherlands/Rotterdam_1364740/2020**	EPI_ISL_413584	2020-03-03	unknown	Erasmus Medical Center	Nieuwenhuijse et al.
**Netherlands/Nootdorp_1364222/2020**	EPI_ISL_413579	2020-03-03	MHC Haaglanden	Erasmus Medical Center	Nieuwenhuijse et al.
**Mexico/CDMX-InDRE_01/2020**	EPI_ISL_412972	2020-02-27	Instituto Nacional de Enfermedades Respiratorias	Instituto de Diagnostico y Referencia Epidemiologicos (INDRE)	Ernesto et al.
**Germany/Baden-Wrttemberg1/2020**	EPI_ISL_412912	2020-02-25	State Health Office Baden-Wuerttemberg	Charité Universitätsmedizin Berlin, Institute of Virology	Corman et al.
**Finland/FIN03032020C/2020**	EPI_ISL_413604	2020-03-03	Department of Virology and Immunology, University of Helsinki and Helsinki University Hospital, Huslab Finland	Department of Virology, Faculty of Medicine, University of Helsinki, Helsinki, Finland	Teemu Smura, Hannimari Kallio-Kokko, Olli Vapalahti
**Wales/PHW1/2020**	EPI_ISL_413555	2020-02-27	Wales Specialist Virology Centre	Public Health Wales Microbiology Cardiff	Moore et al.
**Portugal/CV63/2020**	EPI_ISL_413648	2020-03-01	Centro Hospitalar e Universitário de Sao Joao, Porto	Instituto Nacional de Saude (INSA)	Guiomar et al.
**Netherlands/Zeewolde_1365080/2020**	EPI_ISL_413591	2020-03-02	MHC Flevoland	Erasmus Medical Center	Nieuwenhuijse et al.
**Netherlands/Utrecht_1363628/2020**	EPI_ISL_413589	2020-03-01	MHC Utrecht	Erasmus Medical Center	Nieuwenhuijse et al.
**Netherlands/Haarlem_1363688/2020**	EPI_ISL_413572	2020-03-01	MHC Kennemerland	Erasmus Medical Center	Nieuwenhuijse et al.
**Netherlands/Blaricum_1364780/2020**	EPI_ISL_413566	2020-03-02	MHC Gooi & Vechtstreek	Erasmus Medical Center	Nieuwenhuijse et al.
**Luxembourg/Lux1/2020**	EPI_ISL_413593	2020-02-29	Laboratoire National de Santé	Erasmus Medical Center	Nieuwenhuijse et al.
**Italy/UniSR1/2020**	EPI_ISL_413489	2020-03-03	Laboratorio di Microbiologia e Virologia, Università Vita-Salute San Raffaele, Milano	Laboratorio di Microbiologia e Virologia, Università Vita-Salute San Raffaele, Milano	Diotti et al.
**Italy/CDG1/2020**	EPI_ISL_412973	2020-02-20	Department of Infectious Diseases, Istituto Superiore di Sanità, Roma , Italy	Virology Laboratory, Scientific Department, Army Medical Center	Stefanelli et al.

**Germany/BavPat1/2020**	EPI_ISL_406862	2020-01-28	Charité Universitätsmedizin Berlin, Institute of Virology; Institut für Mikrobiologie der Bundeswehr, Munich	Charité Universitätsmedizin Berlin, Institute of Virology	Corman et al.
**Argentina/C1374/2020**	EPI_ISL_420599	2020-03-18	Servicio Virosis Respiratorias-Departamento Virologia-INEI	Instituto Nacional Enfermedades Infecciosas C.G.Malbran	Baumeister E. et al (https://dx.doi.org/10.1101/2020.05.23.20111443)
**Argentina/PAIS_A012/2020**	EPI_ISL_430803	2020-04-01	Laboratorio de Virologia del Hospital de Niños Dr. Ricardo Gutierrez	Área de Secuenciación del Laboratorio de Virología del Hospital de Niños Dr. Ricardo Gutierrez	Nabaes Jodar et al A (https://dx.doi.org/10.1101/2020.05.23.20111443)
**Argentina/PAIS_A015/2020**	EPI_ISL_430806	2020-04-01	Departamento de Biologia y genetica molecular, IACA Laboratorios.	Área de Secuenciación del Laboratorio de Virología del Hospital de Niños Dr. Ricardo Gutierrez	Nabaes Jodar et al B
**Argentina/PAIS_A009/2020**	EPI_ISL_430801	2020-03-31	Laboratorio de Virologia del Hospital de Niños Dr. Ricardo Gutierrez	Área de Secuenciación del Laboratorio de Virología del Hospital de Niños Dr. Ricardo Gutierrez	Nabaes Jodar et al A (https://dx.doi.org/10.1101/2020.05.23.20111443)
**Argentina/PAIS_A013/2020**	EPI_ISL_430804	2020-04-01	Laboratorio de Virologia del Hospital de Niños Dr. Ricardo Gutierrez	Área de Secuenciación del Laboratorio de Virología del Hospital de Niños Dr. Ricardo Gutierrez	Nabaes Jodar et al A (https://dx.doi.org/10.1101/2020.05.23.20111443)
**Argentina/PAIS_A007/2020**	EPI_ISL_430799	2020-03-30	Laboratorio de Virologia del Hospital de Niños Dr. Ricardo Gutierrez	Área de Secuenciación del Laboratorio de Virología del Hospital de Niños Dr. Ricardo Gutierrez	Nabaes Jodar et al A (https://dx.doi.org/10.1101/2020.05.23.20111443)
**Argentina/PAIS_A004/2020**	EPI_ISL_430796	2020-03-27	Departamento de Biologia y genetica molecular, IACA Laboratorios.	Área de Secuenciación del Laboratorio de Virología del Hospital de Niños Dr. Ricardo Gutierrez	Nabaes Jodar et al A (https://dx.doi.org/10.1101/2020.05.23.20111443)
**Argentina/C3013/2020**	EPI_ISL_420598	2020-03-22	Servicio Virosis Respiratorias-Departamento Virologia-INEI	Instituto Nacional Enfermedades Infecciosas C.G.Malbran	Baumeister E. et al (https://dx.doi.org/10.1101/2020.05.23.20111443)
**Argentina/PAIS_A005/2020**	EPI_ISL_430797	2020-04-03	Departamento de Biologia y genetica molecular, IACA Laboratorios.	Área de Secuenciación del Laboratorio de Virología del Hospital de Niños Dr. Ricardo Gutierrez	Nabaes Jodar et al A (https://dx.doi.org/10.1101/2020.05.23.20111443)

**Argentina/PAIS_A014/2020**	EPI_ISL_430805	2020-04-11	Departamento de Biologia y genetica molecular, IACA Laboratorios.	Área de Secuenciación del Laboratorio de Virología del Hospital de Niños Dr. Ricardo Gutierrez	Nabaes Jodar et al A (https://dx.doi.org/10.1101/2020.05.23.20111443)
**Argentina/C121/2020**	EPI_ISL_420600	2020-04-11	Servicio Virosis Respiratorias-Departamento Virologia-INEI	Instituto Nacional Enfermedades Infecciosas C.G.Malbran	Nabaes Jodar et al A (https://dx.doi.org/10.1101/2020.05.23.20111443)
**Argentina/PAIS_A016/2020**	EPI_ISL_430807	2020-04-11	Laboratorio de Virologia del Hospital de Niños Dr. Ricardo Gutierrez	Área de Secuenciación del Laboratorio de Virología del Hospital de Niños Dr. Ricardo Gutierrez	Nabaes Jodar et al B
**Argentina/PAIS_A011/2020**	EPI_ISL_430802	2020-03-24	Departamento de Biologia y genetica molecular, IACA Laboratorios.	Área de Secuenciación del Laboratorio de Virología del Hospital de Niños Dr. Ricardo Gutierrez	Nabaes Jodar et al B
**Argentina/PAIS_A001/2020**	EPI_ISL_430793	2020-03-27	Laboratorio Análisis Clínicos, Unidad de Servicios Diagnósticos, Swiss Medical Group	Área de Secuenciación del Laboratorio de Virología del Hospital de Niños Dr. Ricardo Gutierrez	Nabaes Jodar et al A (https://dx.doi.org/10.1101/2020.05.23.20111443)
**Argentina/Heritas_HG001/2020**	EPI_ISL_476496	2020-04-01	Hospital Garrahan	Héritas	Nabaes Jodar et al A (https://dx.doi.org/10.1101/2020.05.23.20111443)
**Argentina/Heritas_HG006/2020**	EPI_ISL_476561	2020-04-02	Hospital Garrahan	Héritas	Nabaes Jodar et al A (https://dx.doi.org/10.1101/2020.05.23.20111443)
**Argentina/Heritas_HG007/2020**	EPI_ISL_476565	2020-03-01	Hospital de Pediatria "Prof. Dr. Juan P Garrahan"	Héritas	Nabaes Jodar et al A (https://dx.doi.org/10.1101/2020.05.23.20111443)
**Argentina/PAIS_A023/2020**	EPI_ISL_430814	2020-04-22	Laboratorio de Virologia del Hospital de Niños Dr. Ricardo Gutierrez	Área de Secuenciación del Laboratorio de Virología del Hospital de Niños Dr. Ricardo Gutierrez	Dalmacio Pereyra et al
**Argentina/PAIS_A018/2020**	EPI_ISL_430809	2020-04-18	Laboratorio de Virologia del Hospital de Niños Dr. Ricardo Gutierrez	Área de Secuenciación del Laboratorio de Virología del Hospital de Niños Dr. Ricardo Gutierrez	Nabaes Jodar et al A (https://dx.doi.org/10.1101/2020.05.23.20111443)

**Argentina/PAIS_A017/2020**	EPI_ISL_430808	2020-05-07	Departamento de Biologia y genética molecular, IACA Laboratorios.	Área de Secuenciación del Laboratorio de Virología del Hospital de Niños Dr. Ricardo Gutierrez	Roberta Crespo et al
**Argentina/PAIS_A020/2020**	EPI_ISL_430811	2020-04-18	Laboratorio de Virologia del Hospital de Niños Dr. Ricardo Gutierrez	Área de Secuenciación del Laboratorio de Virología del Hospital de Niños Dr. Ricardo Gutierrez	Nabaes Jodar et al A (https://dx.doi.org/10.1101/2020.05.23.20111443)
**Argentina/PAIS_A021/2020**	EPI_ISL_430812	2020-05-09	Laboratorio de Virologia del Hospital de Niños Dr. Ricardo Gutierrez	Área de Secuenciación del Laboratorio de Virología del Hospital de Niños Dr. Ricardo Gutierrez	Andrea Mangano et al
**Argentina/PAIS_A022/2020**	EPI_ISL_430813	2020-04-17	Laboratorio de Virologia del Hospital de Niños Dr. Ricardo Gutierrez	Área de Secuenciación del Laboratorio de Virología del Hospital de Niños Dr. Ricardo Gutierrez	Nabaes Jodar et al A (https://dx.doi.org/10.1101/2020.05.23.20111443)
**Argentina/Heritas_HG009/2020**	EPI_ISL_476568	2020-04-07	Hospital de Pediatria "Prof. Dr. Juan P Garrahan"	Héritas	Nabaes Jodar et al A (https://dx.doi.org/10.1101/2020.05.23.20111443)
**Argentina/PAIS_A003/2020**	EPI_ISL_430795	2020-05-14	Laboratorio de Virologia del Hospital de Niños Dr. Ricardo Gutierrez	Área de Secuenciación del Laboratorio de Virología del Hospital de Niños Dr. Ricardo Gutierrez	Cristian Rohr et al
**Argentina/PAIS_A019/2020**	EPI_ISL_430810	2020-05-18	Laboratorio de Virologia del Hospital de Niños Dr. Ricardo Gutierrez	Área de Secuenciación del Laboratorio de Virología del Hospital de Niños Dr. Ricardo Gutierrez	Dalmacio Pereyra et al
**Argentina/Heritas_HG015/2020**	EPI_ISL_476567	2020-04-18	Hospital de Pediatria "Prof. Dr. Juan P Garrahan"	Héritas	Nabaes Jodar et al A (https://dx.doi.org/10.1101/2020.05.23.20111443)
**Argentina/PAIS_A025/2020**	EPI_ISL_430816	2020-05-16	Laboratorio de Virologia del Hospital de Niños Dr. Ricardo Gutierrez	Área de Secuenciación del Laboratorio de Virología del Hospital de Niños Dr. Ricardo Gutierrez	Dalmacio Pereyra et al
**Argentina/Heritas_HG014/2020**	EPI_ISL_476563	2020-05-19	Hospital de Pediatria "Prof. Dr. Juan P Garrahan"	Héritas	Dalmacio Pereyra et al
**Argentina/Heritas_HG018/2020**	EPI_ISL_476571	2020-05-22	Hospital de Pediatria "Prof. Dr. Juan P Garrahan"	Héritas	Dalmacio Pereyra et al

**Argentina/Heritas_HG019/2020**	EPI_ISL_476573	2020-03-28	Hospital de Pediatria "Prof. Dr. Juan P Garrahan"	Héritas	Nabaes Jodar et al A (https://dx.doi.org/10.1101/2020.05.23.20111443)
**Argentina/PAIS_A006/2020**	EPI_ISL_430798	2020-03-26	Departamento de Biologia y genetica molecular, IACA Laboratorios.	Área de Secuenciación del Laboratorio de Virología del Hospital de Niños Dr. Ricardo Gutierrez	Nabaes Jodar et al B
**Brazil/AC162535-IEC/2020**	EPI_ISL_458139	2020-03-18	Evandro Chagas Institute	Evandro Chagas Institute	Santos et al (https://dx.doi.org/10.1101/2020.06.17.158006)
**Brazil/AL-837/2020**	EPI_ISL_427292	2020-03-18	LACEN-AL - Laboratorio Central de Alagoas	Instituto Oswaldo Cruz FIOCRUZ - Laboratory of Respiratory Viruses and Measles (LVRS)	Paola Resende et al (https://dx.doi.org/10.1101/2020.06.17.158006)
**Brazil/AP162741-IEC/2020**	EPI_ISL_458138	2020-04-03	Evandro Chagas Institute	Evandro Chagas Institute	Santos et al (https://dx.doi.org/10.1101/2020.06.17.158006)
**Brazil/AP162966-IEC/2020**	EPI_ISL_458142	2020-04-05	Evandro Chagas Institute	Evandro Chagas Institute	Santos et al (https://dx.doi.org/10.1101/2020.06.17.158006)
**Brazil/AP161167-IEC/2020**	EPI_ISL_450873	2020-03-17	Evandro Chagas Institute	Evandro Chagas Institute	Santos et al (https://dx.doi.org/10.1101/2020.06.17.158006)
**Brazil/AP164346-IEC/2020**	EPI_ISL_458145	2020-04-20	Evandro Chagas Institute	Evandro Chagas Institute	Santos et al (https://dx.doi.org/10.1101/2020.06.17.158006)
**Brazil/AP163972-IEC/2020**	EPI_ISL_458144	2020-04-15	Evandro Chagas Institute	Evandro Chagas Institute	Santos et al (https://dx.doi.org/10.1101/2020.06.17.158006)
**Brazil/AP164082-IEC/2020**	EPI_ISL_458143	2020-04-15	Evandro Chagas Institute	Evandro Chagas Institute	Santos et al (https://dx.doi.org/10.1101/2020.06.17.158006)
**Brazil/AM0201/2020**	EPI_ISL_470568	2020-04-01	Hermes Pardini	Bioinformatics Laboratory / LNCC	Alexandra Gerber et al
**Brazil/AM0203/2020**	EPI_ISL_470570	2020-04-02	Hermes Pardini	Bioinformatics Laboratory / LNCC	Alexandra Gerber et al

**Brazil/BA-510/2020**	EPI_ISL_427293	2020-03-06	LACEN-BA - Laboratório Central de Saúde Pública Professor Gonçalo Moniz	Instituto Oswaldo Cruz FIOCRUZ - Laboratory of Respiratory Viruses and Measles (LVRS)	Paola Resende et al (https://dx.doi.org/10.1101/2020.06.17.158006)
**Brazil/BA-312/2020**	EPI_ISL_415105	2020-03-04	Laboratório Central de Saúde Pública Professor Gonçalo Moniz - LACEN/BA	Instituto Oswaldo Cruz FIOCRUZ - Laboratory of Respiratory Viruses and Measles (LVRS)	Paola Resende et al (https://dx.doi.org/10.1101/2020.06.17.158006)
**Brazil/L17_CD359/2020**	EPI_ISL_476305	2020-03-31	DB Diagnósticos do Brasil		Samples: Nelson Gaburo Jr et al
**Brazil/CE0206/2020**	EPI_ISL_470573	2020-04-02	Hermes Pardini	Bioinformatics Laboratory / LNCC	Alexandra Gerber et al
**Brazil/L16_CD346/2020**	EPI_ISL_476289	2020-03-30	DB Diagnósticos do Brasil	Instituto de Medicina Tropical da Universidade de São Paulo	Samples: Nelson Gaburo Jr et al
**Brazil/L19_CD408/2020**	EPI_ISL_476331	2020-04-02	DB Diagnósticos do Brasil	Instituto de Medicina Tropical da Universidade de São Paulo	Samples: Nelson Gaburo Jr et al
**Brazil/L16_CD344/2020**	EPI_ISL_476288	2020-03-30	DB Diagnósticos do Brasil	Instituto de Medicina Tropical da Universidade de São Paulo	Samples: Nelson Gaburo Jr et al
**Brazil/DFBR-0001/2020**	EPI_ISL_426580	2020-03-13	Instituto Sabin	Laboratory of Virology	Fernando L Melo et al (https://dx.doi.org/10.1101/2020.06.11.20128249)
**Brazil/DF-619i/2020**	EPI_ISL_427295	2020-03-13	Instituto Oswaldo Cruz FIOCRUZ - Laboratory of Respiratory Viruses and Measles (LVRS)	Instituto Oswaldo Cruz FIOCRUZ - Laboratory of Respiratory Viruses and Measles (LVRS)	Paola Resende et al (https://dx.doi.org/10.1101/2020.06.17.158006)
**Brazil/DF-615i/2020**	EPI_ISL_427294	2020-03-13	Instituto Oswaldo Cruz FIOCRUZ - Laboratory of Respiratory Viruses and Measles (LVRS)	Instituto Oswaldo Cruz FIOCRUZ - Laboratory of Respiratory Viruses and Measles (LVRS)	Paola Resende et al (https://dx.doi.org/10.1101/2020.06.17.158006)
**Brazil/DF-862/2020**	EPI_ISL_427297	2020-03-23	Instituto Oswaldo Cruz FIOCRUZ - Laboratory of Respiratory Viruses and Measles (LVRS)	Instituto Oswaldo Cruz FIOCRUZ - Laboratory of Respiratory Viruses and Measles (LVRS)	Paola Resende et al (https://dx.doi.org/10.1101/2020.06.17.158006)

**Brazil/DF-891/2020**	EPI_ISL_427298	2020-03-22	Instituto Oswaldo Cruz FIOCRUZ - Laboratory of Respiratory Viruses and Measles (LVRS)	Instituto Oswaldo Cruz FIOCRUZ - Laboratory of Respiratory Viruses and Measles (LVRS)	Paola Resende et al (https://dx.doi.org/10.1101/2020.06.17.158006)
**Brazil/DF-861/2020**	EPI_ISL_427296	2020-03-23	Instituto Oswaldo Cruz FIOCRUZ - Laboratory of Respiratory Viruses and Measles (LVRS)	Instituto Oswaldo Cruz FIOCRUZ - Laboratory of Respiratory Viruses and Measles (LVRS)	Paola Resende et al (https://dx.doi.org/10.1101/2020.06.17.158006)
**Brazil/GO0209/2020**	EPI_ISL_470576	2020-04-03	Hermes Pardini	Bioinformatics Laboratory / LNCC	Alexandra Gerber et al
**Brazil/GO0210/2020**	EPI_ISL_470577	2020-04-04	Hermes Pardini	Bioinformatics Laboratory / LNCC	Alexandra Gerber et al
**Brazil/L19_CD413/2020**	EPI_ISL_476336	2020-04-20	DB Diagnósticos do Brasil	Instituto de Medicina Tropical da Universidade de São Paulo	Gaburo Jr et al.
**Brazil/MA163069-IEC/2020**	EPI_ISL_458149	2020-04-06	Evandro Chagas Institute	Evandro Chagas Institute	Santos et al (https://dx.doi.org/10.1101/2020.06.17.158006)
**Brazil/L19_CD403/2020**	EPI_ISL_476327	2020-04-02	DB Diagnósticos do Brasil	Instituto de Medicina Tropical da Universidade de São Paulo	Samples: Nelson Gaburo Jr et al
**Brazil/L17_CD363/2020**	EPI_ISL_476309	2020-03-31	DB Diagnósticos do Brasil	Instituto de Medicina Tropical da Universidade de São Paulo	Samples: Nelson Gaburo Jr et al
**Brazil/CV12/2020**	EPI_ISL_429674	2020-03-11	Central Public Health Laboratory/Octávio Magalhães Institute (IOM) from the Ezequiel Dias Foundation (FUNED)	Instituto Octávio Magalhães / Fundação Ezequiel Dias (IOM/Funed)	Talita Adelino et al (https://dx.doi.org/10.1101/2020.05.05.20091611)
**Brazil/CV21/2020**	EPI_ISL_429681	2020-03-16	Central Public Health Laboratory/Octávio Magalhães Institute (IOM) from the Ezequiel Dias Foundation (FUNED)	Instituto Octávio Magalhães / Fundação Ezequiel Dias (IOM/Funed)	Talita Adelino et al (https://dx.doi.org/10.1101/2020.05.05.20091611)
**Brazil/CV6/2020**	EPI_ISL_429669	2020-03-13	Central Public Health Laboratory/Octávio Magalhães Institute (IOM)	Instituto Octávio Magalhães / Fundação Ezequiel Dias (IOM/Funed)	Talita Adelino et al (https://dx.doi.org/10.1101/2020.05.05.20091611)

			from the Ezequiel Dias Foundation (FUNED)		
**Brazil/CV4/2020**	EPI_ISL_429667	2020-03-09	Central Public Health Laboratory/Octávio Magalhães Institute (IOM) from the Ezequiel Dias Foundation (FUNED)	Instituto Octávio Magalhães / Fundação Ezequiel Dias (IOM/Funed)	Talita Adelino et al (https://dx.doi.org/10.1101/2020.05.05.20091611)
**Brazil/CV31/2020**	EPI_ISL_429687	2020-03-17	Central Public Health Laboratory/Octávio Magalhães Institute (IOM) from the Ezequiel Dias Foundation (FUNED)	Instituto Octávio Magalhães / Fundação Ezequiel Dias (IOM/Funed)	Talita Adelino et al (https://dx.doi.org/10.1101/2020.05.05.20091611)
**Brazil/MG0288/2020**	EPI_ISL_470593	2020-04-09	Simile	Bioinformatics Laboratory / LNCC	Alexandra Gerber et al
**Brazil/CV42/2020**	EPI_ISL_429695	2020-03-20	Central Public Health Laboratory/Octávio Magalhães Institute (IOM) from the Ezequiel Dias Foundation (FUNED)	Instituto Octávio Magalhães / Fundação Ezequiel Dias (IOM/Funed)	Talita Adelino et al (https://dx.doi.org/10.1101/2020.05.05.20091611)
**Brazil/CV49/2020**	EPI_ISL_429702	2020-03-22	Central Public Health Laboratory/Octávio Magalhães Institute (IOM) from the Ezequiel Dias Foundation (FUNED)	Instituto Octávio Magalhães / Fundação Ezequiel Dias (IOM/Funed)	Talita Adelino et al (https://dx.doi.org/10.1101/2020.05.05.20091611)
**Brazil/CV16/2020**	EPI_ISL_429676	2020-03-17	Central Public Health Laboratory/Octávio Magalhães Institute (IOM) from the Ezequiel Dias Foundation (FUNED)	Instituto Octávio Magalhães / Fundação Ezequiel Dias (IOM/Funed)	Talita Adelino et al (https://dx.doi.org/10.1101/2020.05.05.20091611)
**Brazil/CV8/2020**	EPI_ISL_429671	2020-03-17	Central Public Health Laboratory/Octávio Magalhães Institute (IOM) from the Ezequiel Dias Foundation (FUNED)	Instituto Octávio Magalhães / Fundação Ezequiel Dias (IOM/Funed)	Filipe Romero et al
**Brazil/MG0220/2020**	EPI_ISL_470586	2020-03-17	Hermes Pardini	Bioinformatics Laboratory / LNCC	Alexandra Gerber et al
**Brazil/MG0216/2020**	EPI_ISL_470582	2020-03-17	Hermes Pardini	Bioinformatics Laboratory / LNCC	Filipe Romero et al

**Brazil/CV32/2020**	EPI_ISL_429688	2020-03-17	Central Public Health Laboratory/Octávio Magalhães Institute (IOM) from the Ezequiel Dias Foundation (FUNED)	Instituto Octávio Magalhães / Fundação Ezequiel Dias (IOM/Funed)	Talita Adelino et al (https://dx.doi.org/10.1101/2020.05.05.20091611)
**Brazil/MG0286/2020**	EPI_ISL_470591	2020-04-17	Simile	Bioinformatics Laboratory / LNCC	Alexandra Gerber et al
**Brazil/MG0222/2020**	EPI_ISL_470588	2020-04-06	Hermes Pardini	Bioinformatics Laboratory / LNCC	Alexandra Gerber et al
**Brazil/MG0289/2020**	EPI_ISL_470594	2020-04-08	Simile	Bioinformatics Laboratory / LNCC	Alexandra Gerber et al
**Brazil/MG0218/2020**	EPI_ISL_470584	2020-03-23	Hermes Pardini	Bioinformatics Laboratory / LNCC	Alexandra Gerber et al
**Brazil/MG0219/2020**	EPI_ISL_470585	2020-04-03	Hermes Pardini	Bioinformatics Laboratory / LNCC	Alexandra Gerber et al
**Brazil/MG0215/2020**	EPI_ISL_470581	2020-03-19	Hermes Pardini	Bioinformatics Laboratory / LNCC	Alexandra Gerber et al
**Brazil/PA0226/2020**	EPI_ISL_470600	2020-04-01	Hermes Pardini	Bioinformatics Laboratory / LNCC	Alexandra Gerber et al
**Brazil/PA0227/2020**	EPI_ISL_470601	2020-04-01	Hermes Pardini	Bioinformatics Laboratory / LNCC	Alexandra Gerber et al
**Brazil/PA0229/2020**	EPI_ISL_470603	2020-04-01	Hermes Pardini	Bioinformatics Laboratory / LNCC	Alexandra Gerber et al
**Brazil/PA0231/2020**	EPI_ISL_470605	2020-04-02	Hermes Pardini	Bioinformatics Laboratory / LNCC	Alexandra Gerber et al
**Brazil/PA0236/2020**	EPI_ISL_470610	2020-04-03	Hermes Pardini	Bioinformatics Laboratory / LNCC	Alexandra Gerber et al
**Brazil/PA161548-IEC/2020**	EPI_ISL_450874	2020-03-20	Evandro Chagas Institute	Evandro Chagas Institute	Santos et al (https://dx.doi.org/10.1101/2020.06.17.158006)
**Brazil/PA162802-IEC/2020**	EPI_ISL_458140	2020-04-07	Evandro Chagas Institute	Evandro Chagas Institute	Santos et al (https://dx.doi.org/10.1101/2020.06.17.158006)
**Brazil/PA164173-IEC/2020**	EPI_ISL_458146	2020-04-23	Evandro Chagas Institute	Evandro Chagas Institute	Santos et al (https://dx.doi.org/10.1101/2020.06.17.158006)

**Brazil/PA164218-IEC/2020**	EPI_ISL_458147	2020-04-24	Evandro Chagas Institute	Evandro Chagas Institute	Santos et al (https://dx.doi.org/10.1101/2020.06.17.158006)
**Brazil/PA164239-IEC/2020**	EPI_ISL_458141	2020-04-26	Evandro Chagas Institute	Evandro Chagas Institute	Santos et al (https://dx.doi.org/10.1101/2020.06.17.158006)
**Brazil/PA164684-IEC/2020**	EPI_ISL_458148	2020-04-27	Evandro Chagas Institute	Evandro Chagas Institute	Santos et al (https://dx.doi.org/10.1101/2020.06.17.158006)
**Brazil/L16_CD333/2020**	EPI_ISL_476282	2020-03-28	DB Diagnósticos do Brasil	Instituto de Medicina Tropical da Universidade de São Paulo	Samples: Nelson Gaburo Jr et al
**Brazil/L17_CD366/2020**	EPI_ISL_476312	2020-03-31	DB Diagnósticos do Brasil	Instituto de Medicina Tropical da Universidade de São Paulo	Samples: Nelson Gaburo Jr et al
**Brazil/L19_CD389/2020**	EPI_ISL_476318	2020-04-01	DB Diagnósticos do Brasil	Instituto de Medicina Tropical da Universidade de São Paulo	Samples: Nelson Gaburo Jr et al
**Brazil/L17_CD365/2020**	EPI_ISL_476311	2020-03-31	DB Diagnósticos do Brasil	Instituto de Medicina Tropical da Universidade de São Paulo	Samples: Nelson Gaburo Jr et al
**Brazil/L20_CD432/2020**	EPI_ISL_476359	2020-04-03	DB Diagnósticos do Brasil	Instituto de Medicina Tropical da Universidade de São Paulo	Samples: Nelson Gaburo Jr et al
**Brazil/RJ-2676/2020**	EPI_ISL_467354	2020-04-24	Laboratory of Respiratory Viruses and Measles, Oswaldo Cruz Institute, FIOCRUZ	Laboratory of Respiratory Viruses and Measles, Oswaldo Cruz Institute, FIOCRUZ	Paola Resende et al (https://dx.doi.org/10.1101/2020.06.17.158006)
**Brazil/RJ0248/2020**	EPI_ISL_470616	2020-03-24	Laboratorio de Virologia Molecular / UFRJ	Bioinformatics Laboratory / LNCC	Alexandra Gerber et al
**Brazil/RJ0251/2020**	EPI_ISL_470619	2020-03-27	Laboratorio de Virologia Molecular / UFRJ	Bioinformatics Laboratory / LNCC	Alexandra Gerber et al
**Brazil/RJ-314/2020**	EPI_ISL_414045	2020-03-04	LACEN RJ - Laboratório Central de Saúde Pública Noel Nutels	Instituto Oswaldo Cruz FIOCRUZ - Laboratory of Respiratory Viruses and Measles (LVRS)	Paola Resende et al (https://dx.doi.org/10.1101/2020.06.17.158006)

**Brazil/RJ-1111/2020**	EPI_ISL_456076	2020-04-02	LACEN RJ - Laboratório Central de Saúde Pública Noel Nutels	Laboratory of Respiratory Viruses and Measles, Oswaldo Cruz Institute, FIOCRUZ	Paola Resende et al (https://dx.doi.org/10.1101/2020.06.17.158006)
**Brazil/RJ-1466/2020**	EPI_ISL_456081	2020-04-24	Laboratory of Respiratory Viruses and Measles, Oswaldo Cruz Institute, FIOCRUZ	Laboratory of Respiratory Viruses and Measles, Oswaldo Cruz Institute, FIOCRUZ	Alexandra Gerber et al
**Brazil/RJ-1927/2020**	EPI_ISL_456093	2020-03-25	Laboratory of Respiratory Viruses and Measles, Oswaldo Cruz Institute, FIOCRUZ	Laboratory of Respiratory Viruses and Measles, Oswaldo Cruz Institute, FIOCRUZ	Paola Resende et al (https://dx.doi.org/10.1101/2020.06.17.158006)
**Brazil/RJ-2195/2020**	EPI_ISL_467348	2020-04-03	Laboratory of Respiratory Viruses and Measles, Oswaldo Cruz Institute, FIOCRUZ	Laboratory of Respiratory Viruses and Measles, Oswaldo Cruz Institute, FIOCRUZ	Paola Resende et al (https://dx.doi.org/10.1101/2020.06.17.158006)
**Brazil/RJ-2422/2020**	EPI_ISL_467352	2020-04-13	Laboratory of Respiratory Viruses and Measles, Oswaldo Cruz Institute, FIOCRUZ	Laboratory of Respiratory Viruses and Measles, Oswaldo Cruz Institute, FIOCRUZ	Paola Resende et al (https://dx.doi.org/10.1101/2020.06.17.158006)
**Brazil/RJ-2669/2020**	EPI_ISL_467353	2020-04-27	Laboratory of Respiratory Viruses and Measles, Oswaldo Cruz Institute, FIOCRUZ	Laboratory of Respiratory Viruses and Measles, Oswaldo Cruz Institute, FIOCRUZ	Paola Resende et al (https://dx.doi.org/10.1101/2020.06.17.158006)
**Brazil/RJ-2868/2020**	EPI_ISL_467371	2020-04-24	Laboratory of Respiratory Viruses and Measles, Oswaldo Cruz Institute, FIOCRUZ	Laboratory of Respiratory Viruses and Measles, Oswaldo Cruz Institute, FIOCRUZ	Alexandra Gerber et al
**Brazil/RJ-477/2020**	EPI_ISL_427300	2020-04-13	Instituto Oswaldo Cruz FIOCRUZ - Laboratory of Respiratory Viruses and Measles (LVRS)	Instituto Oswaldo Cruz FIOCRUZ - Laboratory of Respiratory Viruses and Measles (LVRS)	Paola Resende et al (https://dx.doi.org/10.1101/2020.06.17.158006)
**Brazil/RJ-763/2020**	EPI_ISL_427302	2020-04-16	Instituto Oswaldo Cruz FIOCRUZ - Laboratory of Respiratory Viruses and Measles (LVRS)	Instituto Oswaldo Cruz FIOCRUZ - Laboratory of Respiratory Viruses and Measles (LVRS)	Paola Resende et al (https://dx.doi.org/10.1101/2020.06.17.158006)

**Brazil/RJ-899/2020**	EPI_ISL_456071	2020-04-06	Laboratory of Respiratory Viruses and Measles, Oswaldo Cruz Institute, FIOCRUZ	Laboratory of Respiratory Viruses and Measles, Oswaldo Cruz Institute, FIOCRUZ	Paola Resende et al (https://dx.doi.org/10.1101/2020.06.17.158006)
**Brazil/L19_CD390/2020**	EPI_ISL_476319	2020-04-01	DB Diagnósticos do Brasil	Instituto de Medicina Tropical da Universidade de São Paulo	Samples: Nelson Gaburo Jr et al
**Brazil/L15_CD265/2020**	EPI_ISL_476259	2020-03-29	Hospital das Clínicas da Faculdade de Medicina da Universidade de São Paulo	Instituto de Medicina Tropical da Universidade de São Paulo	Samples: Ingra Morales Claro et al
**Brazil/L16_CD314/2020**	EPI_ISL_476278	2020-03-24	DB Diagnósticos do Brasil	Instituto de Medicina Tropical da Universidade de São Paulo	Samples: Nelson Gaburo Jr et al
**Brazil/L16_CD350/2020**	EPI_ISL_476292	2020-03-30	DB Diagnósticos do Brasil	Instituto de Medicina Tropical da Universidade de São Paulo	Samples: Nelson Gaburo Jr et al
**Brazil/L17_CD322/2020**	EPI_ISL_476297	2020-03-26	DB Diagnósticos do Brasil	Instituto de Medicina Tropical da Universidade de São Paulo	Samples: Nelson Gaburo Jr et al
**Brazil/L19_CD399/2020**	EPI_ISL_476324	2020-04-01	DB Diagnósticos do Brasil	Instituto de Medicina Tropical da Universidade de São Paulo	Samples: Nelson Gaburo Jr et al
**Brazil/SC0244/2020**	EPI_ISL_470653	2020-03-18	Hermes Pardini	Bioinformatics Laboratory / LNCC	Alexandra Gerber et al
**Brazil/SC0245/2020**	EPI_ISL_470654	2020-03-18	Hermes Pardini	Bioinformatics Laboratory / LNCC	Alexandra Gerber et al
**Brazil/SC0246/2020**	EPI_ISL_470655	2020-04-03	Hermes Pardini	Bioinformatics Laboratory / LNCC	Alexandra Gerber et al
**Brazil/SC-769/2020**	EPI_ISL_427306	2020-03-10	LACEN-SC - Laboratorio Central de Santa Catarina	Instituto Oswaldo Cruz FIOCRUZ - Laboratory of Respiratory Viruses and Measles (LVRS)	Paola Resende et al (https://dx.doi.org/10.1101/2020.06.17.158006)
**Brazil/SP-139/2020**	EPI_ISL_468306	2020-03-20	Laboratorio Municipal de Analises Clinicas	Instituto Adolfo Lutz, Interdiciplinary Procedures Center, Strategic Laboratory	Claudio Tavares Sacchi, Claudia Regina Gonçalves, Erica Valessa Ramos Gomes

**Brazil/SP-01/2020**	EPI_ISL_414016	2020-02-29	Hospital SãoJoaquim Beneficencia Portuguesa	Instituto Adolfo Lutz, Interdiciplinary Procedures Center, Strategic Laboratory	Claudio Tavares Sacchi et al B (https://dx.doi.org/10.1590/s1678-9946202062030)
**Brazil/SP-06/2020**	EPI_ISL_476320	2020-04-01	DB Diagnósticos do Brasil	Instituto de Medicina Tropical da Universidade de São Paulo	Samples: Nelson Gaburo Jr et al
**Brazil/L19_CD391/2020**	**EPI_ISL_476298**	**2020-03-27**	**DB Diagnósticos do Brasil**	**Instituto de Medicina Tropical da Universidade de São Paulo**	**Samples: Nelson Gaburo Jr et al**
**Brazil/SP-03/2020**	EPI_ISL_416028	2020-03-03	National Influenza Center - Instituto Adolfo Lutz	Instituto Adolfo Lutz, Interdiciplinary Procedures Center, Strategic Laboratory	Claudio Tavares Sacchi et al A (https://dx.doi.org/10.1101/2020.05.23.20111443)
**Brazil/SP-07/2020**	EPI_ISL_416034	2020-03-04	Hospital Israelita Albert Einstein	Instituto Adolfo Lutz, Interdiciplinary Procedures Center, Strategic Laboratory	Claudio Tavares Sacchi et al A (https://dx.doi.org/10.1101/2020.05.23.20111443)
**Brazil/SP-12/2020**	EPI_ISL_476461	2020-03-23	Hospital da Clínicas da Faculdade de Medicina da Universidade de São Paulo	Instituto de Medicina Tropical da Universidade de São Paulo	Samples: Ingra Morales Claro et al
**Brazil/SP-138/2020**	EPI_ISL_416036	2020-03-05	National Influenza Center - Instituto Adolfo Lutz	Instituto Adolfo Lutz, Interdiciplinary Procedures Center, Strategic Laboratory	Claudio Tavares Sacchi et al C
**Brazil/SP-14/2020**	EPI_ISL_471542	2020-04-03	Secretaria de Saude de Mogi das Cruzes	Instituto Adolfo Lutz, Interdiciplinary Procedures Center, Strategic Laboratory	Claudio Tavares Sacchi et al C
**Brazil/SP-127/2020**	EPI_ISL_476471	2020-03-17	Hospital da Clínicas da Faculdade de Medicina da Universidade de São Paulo	Instituto de Medicina Tropical da Universidade de São Paulo	Samples: Ingra Morales Claro et al
**Brazil/SP-505/2020**	EPI_ISL_476410	2020-04-28	Laboratorio de Patologia ClÃ-nica - UNICAMP	Laboratorio de Estudos de Vírus Emergentes - UNICAMP	José Luiz Proença-Modena et al
**Brazil/SP-146/2020**	EPI_ISL_471545	2020-03-22	Hospital Sao Paulo de Ensino da Unifesp	Instituto Adolfo Lutz, Interdiciplinary Procedures Center, Strategic Laboratory	Claudio Tavares Sacchi et al C

**Brazil/SP-537/2020**	EPI_ISL_476441	2020-03-25	Hospital da Clínicas da Faculdade de Medicina da Universidade de São Paulo	Instituto de Medicina Tropical da Universidade de São Paulo	Samples: Ingra Morales Claro et al
**Brazil/SP-516/2020**	EPI_ISL_476417	2020-04-23	Laboratorio de Patologia Clínica - UNICAMP	Laboratorio de Estudos de Vírus Emergentes - UNICAMP	José Luiz Proença-Modena et al
**Brazil/SP-527/2020**	EPI_ISL_471549	2020-04-13	Hospital Municipal Carmen Prudente	Instituto Adolfo Lutz, Interdiciplinary Procedures Center, Strategic Laboratory	Claudio Tavares Sacchi et al C
**Brazil/SP-607/2020**	EPI_ISL_476162	2020-04-09	Laboratorio de Patologia Clínica - UNICAMP	Laboratorio de Estudos de Vírus Emergentes - UNICAMP	José Luiz Proença-Modena et al
**Brazil/SP-508/2020**	EPI_ISL_476168	2020-04-02	Laboratorio de Patologia Clínica - UNICAMP	Laboratorio de Estudos de Vírus Emergentes - UNICAMP	José Luiz Proença-Modena et al
**Chile/Santiago_79/2020**	EPI_ISL_445376	2020-04-04	HOSPITAL SAN JUAN DE DIOS	Instituto de Salud Publica de Chile	Castillo et al.
**Chile/Coyhaique_1/2020**	EPI_ISL_445296	2020-03-21	HOSPITAL REGIONAL DE COYHAIQUE	Instituto de Salud Publica de Chile	Andrés E Castillo et al
**Chile/Puerto_Montt_1/2020**	EPI_ISL_445246	2020-03-05	HOSPITAL PUERTO MONTT	Instituto de Salud Publica de Chile	Andrés E Castillo et al
**Chile/Puerto_Montt_2/2020**	EPI_ISL_445334	2020-03-29	CLINICA UNIVERSITARIA DE PUERTO MONTT S.A.	Instituto de Salud Publica de Chile	Andrés E Castillo et al
**Chile/Puerto_Montt_3/2020**	EPI_ISL_445335	2020-03-29	HOSPITAL DE CALBUCO	Instituto de Salud Publica de Chile	Andrés E Castillo et al
**Chile/Punta_Arenas_1/2020**	EPI_ISL_445268	2020-03-14	HOSPITAL REG.LAUTARO NAVARRO AVARIA	Instituto de Salud Publica de Chile	Andrés E Castillo et al
**Chile/Punta_Arenas_3/2020**	EPI_ISL_445280	2020-03-17	HOSPITAL REG.LAUTARO NAVARRO AVARIA	Instituto de Salud Publica de Chile	Andrés E Castillo et al
**Chile/Rancagua_7/2020**	EPI_ISL_445297	2020-03-21	CLINICA INTEGRAL S.A.	Instituto de Salud Publica de Chile	Andrés E Castillo et al
**Chile/Santiago_10/2020**	EPI_ISL_445253	2020-03-10	CLINICA ALEMANA DE SANTIAGO S.A.	Instituto de Salud Publica de Chile	Andrés E Castillo et al
**Chile/Santiago_49/2020**	EPI_ISL_445328	2020-03-23	MEGASALUD S.A.	Instituto de Salud Publica de Chile	Andrés E Castillo et al
**Chile/Santiago_61/2020**	EPI_ISL_445357	2020-03-12	INTEGRAMEDICA CENTROS MEDICOS S.A.	Instituto de Salud Publica de Chile	Andrés E Castillo et al

**Chile/Santiago_81/2020**	EPI_ISL_445379	2020-04-06	IMALAB- HOSPITAL FACH	Instituto de Salud Publica de Chile	Andrés E Castillo et al
**Chile/Santiago-05015/2020**	EPI_ISL_468751	2020-04-29	Facultad de Medicina UC	Center for Mathematical Modeling and Center for Genome Regulation. Santiago, Chile	Gaete A et al
**Chile/Temuco_11/2020**	EPI_ISL_445345	2020-03-27	HOSPITAL DR.HERNAN HENRIQUEZ ARAVENA	Instituto de Salud Publica de Chile	Andrés E Castillo et al
**Chile/Temuco_13/2020**	EPI_ISL_445347	2020-03-25	HOSPITAL DR.HERNAN HENRIQUEZ ARAVENA	Instituto de Salud Publica de Chile	Andrés E Castillo et al
**Chile/Temuco_2/2020**	EPI_ISL_445279	2020-03-16	LABORATORIO INMUNOLAB SPA	Instituto de Salud Publica de Chile	Andrés E Castillo et al
**Chile/Temuco_6/2020**	EPI_ISL_445341	2020-03-23	HOSPITAL DR.HERNAN HENRIQUEZ ARAVENA	Instituto de Salud Publica de Chile	Andrés E Castillo et al
**Chile/Temuco_9/2020**	EPI_ISL_447119	2020-03-26	HOSPITAL DR.HERNAN HENRIQUEZ ARAVENA	Instituto de Salud Publica de Chile	Andrés E Castillo et al
**Chile/Valparaiso_1/2020**	EPI_ISL_445272	2020-03-15	CLINICA CIUDAD DEL MAR	Instituto de Salud Publica de Chile	Andrés E Castillo et al
**Chile/Valparaiso_2/2020**	EPI_ISL_445285	2020-03-14	CLINICA CIUDAD DEL MAR	Instituto de Salud Publica de Chile	Andrés E Castillo et al
**Chile/Chillan_3/2020**	EPI_ISL_445332	2020-04-29	HOSPITAL HERMINDA MARTIN CHILLAN	Instituto de Salud Publica de Chile	Gaete A et al
**Chile/Concepcion_2/2020**	EPI_ISL_445333	2020-03-16	LABORATORIO CLINICA UNIVERSITARIA DE CONCEPCION	Instituto de Salud Publica de Chile	Andrés E Castillo et al
**Chile/Huasco-00013/2020**	EPI_ISL_468753	2020-03-12	Laboratorio de Biologia Molecular, Facultad de Medicina, Universidad de Atacama	Center for Mathematical Modeling and Center for Genome Regulation. Santiago, Chile	Andrés E Castillo et al
**Chile/Independencia-27991/2020**	EPI_ISL_459857	2020-03-11	Center for Genome Regulation (CRG)	Center for Mathematical Modeling and Center for Genome Regulation. Santiago, Chile	Andrés E Castillo et al
**Chile/Rancagua_1/2020**	EPI_ISL_445273	2020-03-16	LABORATORIO TORRE MEDICA LTDA.	Instituto de Salud Publica de Chile	Andrés E Castillo et al
**Chile/Rancagua_6/2020**	EPI_ISL_445278	2020-03-17	LABORATORIO TORRE MEDICA LTDA.	Instituto de Salud Publica de Chile	Andrés E Castillo et al

**Chile/Santiago_5/2020**	EPI_ISL_445248	2020-03-16	CLINICA ALEMANA DE SANTIAGO S.A.	Instituto de Salud Publica de Chile	Andrés E Castillo et al
**Chile/Santiago_70/2020**	EPI_ISL_445366	2020-03-17	HOSPITAL DR.SOTERO DEL RIO	Instituto de Salud Publica de Chile	Andrés E Castillo et al
**Chile/Santiago-05230/2020**	EPI_ISL_468747	2020-04-30	Facultad de Medicina UC	Center for Mathematical Modeling and Center for Genome Regulation. Santiago, Chile	Gaete A et al
**Chile/Santiago_26/2020**	EPI_ISL_445305	2020-03-16	HOSPITAL DE CARABINEROS	Instituto de Salud Publica de Chile	Andrés E Castillo et al
**Chile/Santiago_21/2020**	EPI_ISL_445264	2020-03-12	CLINICA REDSALUD VITACURA.	Instituto de Salud Publica de Chile	Andrés E Castillo et al
**Chile/Santiago_12/2020**	EPI_ISL_445255	2020-03-10	CLINICA ALEMANA DE SANTIAGO S.A.	Instituto de Salud Publica de Chile	Andrés E Castillo et al
**Chile/Chillan_2/2020**	EPI_ISL_445331	2020-03-12	HOSPITAL HERMINDA MARTIN CHILLAN	Instituto de Salud Publica de Chile	Andrés E Castillo et al
**Chile/Chillan_1/2020**	EPI_ISL_445271	2020-03-13	LABORATORIO CLINICA CHILLAN	Instituto de Salud Publica de Chile	Andrés E Castillo et al
**Chile/Chillan-25338/2020**	EPI_ISL_468760	2020-03-15	Center for Genome Regulation (CRG)	Center for Mathematical Modeling and Center for Genome Regulation. Santiago, Chile	Gaete A et al
**Chile/Concepcion_1/2020**	EPI_ISL_445281	2020-03-17	HOSPITAL CLINICO DEL SUR	Instituto de Salud Publica de Chile	Andrés E Castillo et al
**Chile/Chillan_4/2020**	EPI_ISL_445378	2020-04-04	HOSPITAL DE BULNES	Instituto de Salud Publica de Chile	Andrés E Castillo et al
**Chile/Temuco_1/2020**	EPI_ISL_445270	2020-03-14	HOSPITAL DR.HERNAN HENRIQUEZ ARAVENA	Instituto de Salud Publica de Chile	Andrés E Castillo et al
**Chile/Rancagua_4/2020**	EPI_ISL_445276	2020-03-15	HOSPITAL CLINICO FUSAT	Instituto de Salud Publica de Chile	Andrés E Castillo et al
**Chile/Rancagua_5/2020**	EPI_ISL_445277	2020-03-16	FUNDACION DE SALUD EL TENIENTE	Instituto de Salud Publica de Chile	Andrés E Castillo et al
**Chile/Rancagua_3/2020**	EPI_ISL_445275	2020-03-15	HOSPITAL CLINICO FUSAT	Instituto de Salud Publica de Chile	Andrés E Castillo et al
**Chile/Santiago_13/2020**	EPI_ISL_445256	2020-03-10	CLINICA LAS CONDES S.A.	Instituto de Salud Publica de Chile	Andrés E Castillo et al
**Chile/Santiago_54/2020**	EPI_ISL_445350	2020-04-01	HOSPITAL SAN JUAN DE DIOS	Instituto de Salud Publica de Chile	Andrés E Castillo et al

**Chile/Santiago_77/2020**	EPI_ISL_445374	2020-04-04	HOSPITAL SAN JUAN DE DIOS	Instituto de Salud Publica de Chile	Andrés E Castillo et al
**Chile/Santiago_78/2020**	EPI_ISL_445375	2020-04-04	HOSPITAL SAN JUAN DE DIOS	Instituto de Salud Publica de Chile	Andrés E Castillo et al
**Chile/Santiago_80/2020**	EPI_ISL_445377	2020-04-05	HOSPITAL SAN JUAN DE DIOS	Instituto de Salud Publica de Chile	Andrés E Castillo et al
**Chile/Copiapo-00063/2020**	EPI_ISL_468758	2020-05-01	Laboratorio de Biologia Molecular, Facultad de Medicina, Universidad de Atacama	Center for Mathematical Modeling and Center for Genome Regulation. Santiago, Chile	Gaete A et al
**Chile/Copiapo-00064/2020**	EPI_ISL_468759	2020-05-01	Laboratorio de Biologia Molecular, Facultad de Medicina, Universidad de Atacama	Center for Mathematical Modeling and Center for Genome Regulation. Santiago, Chile	Gaete A et al
**Chile/Santiago-05426/2020**	EPI_ISL_468750	2020-04-30	Facultad de Medicina UC	Center for Mathematical Modeling and Center for Genome Regulation. Santiago, Chile	Gaete A et al
**Chile/Santiago_40/2020**	EPI_ISL_445319	2020-03-17	HOSPITAL FELIX BULNES	Instituto de Salud Publica de Chile	Andrés E Castillo et al
**Chile/Copiapo-00015/2020**	EPI_ISL_468757	2020-04-30	Laboratorio de Biologia Molecular, Facultad de Medicina, Universidad de Atacama	Center for Mathematical Modeling and Center for Genome Regulation. Santiago, Chile	Gaete A et al
**Chile/Copiapo-00007/2020**	EPI_ISL_468755	2020-04-29	Laboratorio de Biologia Molecular, Facultad de Medicina, Universidad de Atacama	Center for Mathematical Modeling and Center for Genome Regulation. Santiago, Chile	Gaete A et al
**Chile/VinaDelMar-28875/2020**	EPI_ISL_459864	2020-03-19	Center for Genome Regulation (CRG)	Center for Mathematical Modeling and Center for Genome Regulation. Santiago, Chile	Gaete A et al
**Chile/Santiago_52/2020**	EPI_ISL_445349	2020-04-01	HOSPITAL SAN JUAN DE DIOS	Instituto de Salud Publica de Chile	Andrés E Castillo et al
**Chile/Valparaiso_3/2020**	EPI_ISL_445286	2020-03-19	HOSPITAL HANGA ROA	Instituto de Salud Publica de Chile	Andrés E Castillo et al
**Chile/Valparaiso_5/2020**	EPI_ISL_445337	2020-03-30	HOSPITAL HANGA ROA	Instituto de Salud Publica de Chile	Andrés E Castillo et al

**Chile/Punta_Arenas_7/2020**	EPI_ISL_445288	2020-03-20	HOSPITAL REG.LAUTARO NAVARRO AVARIA	Instituto de Salud Publica de Chile	Andrés E Castillo et al
**Chile/Punta_Arenas_9/2020**	EPI_ISL_445290	2020-03-21	CLINICA MAGALLANES S.A.	Instituto de Salud Publica de Chile	Andrés E Castillo et al
**Chile/Punta_Arenas_17/2020**	EPI_ISL_445372	2020-03-30	HOSPITAL FF.AA. "CIRUJANO C. GUZMAN	Instituto de Salud Publica de Chile	Andrés E Castillo et al
**Chile/PuertoNatales-31600/2020**	EPI_ISL_459861	2020-03-25	Center for Genome Regulation (CRG)	Center for Mathematical Modeling and Center for Genome Regulation. Santiago, Chile	Gaete A et al
**Colombia/GUR-0206/2020**	EPI_ISL_447738	2020-03-26	Grupo de Investigaciones Microbiológicas-UR (GIMUR), Departamento de Biologia, Facultad de Ciencias Naturales, Universidad del Rosario, Bogotá, Colombia	Grupo de Investigaciones Microbiológicas-UR (GIMUR), Departamento de Biologia, Facultad de Ciencias Naturales, Universidad del Rosario, Bogotá, Colombia Instituto Nacional de Salud, Bogotá, Colombia Icahn School of Medicine at Mount Sinai, New York, USA	Juan David Ramirez et al (https://www.medrxiv.org/content/10.1101/2020.06.11.20125799v1)
**Colombia/GUR-0207/2020**	EPI_ISL_447739	2020-03-27	Grupo de Investigaciones Microbiológicas-UR (GIMUR), Departamento de Biologia, Facultad de Ciencias Naturales, Universidad del Rosario, Bogotá, Colombia	Grupo de Investigaciones Microbiológicas-UR (GIMUR), Departamento de Biologia, Facultad de Ciencias Naturales, Universidad del Rosario, Bogotá, Colombia Instituto Nacional de Salud, Bogotá, Colombia Icahn School of Medicine at Mount Sinai, New York, USA	Juan David Ramirez et al (https://www.medrxiv.org/content/10.1101/2020.06.11.20125799v1)
**Colombia/GUR-0210/2020**	EPI_ISL_447741	2020-03-28	Grupo de Investigaciones Microbiológicas-UR (GIMUR), Departamento de Biologia, Facultad de Ciencias Naturales,	Grupo de Investigaciones Microbiológicas-UR (GIMUR), Departamento de Biologia, Facultad de Ciencias Naturales, Universidad del Rosario,	Juan David Ramirez et al (https://www.medrxiv.org/content/10.1101/2020.06.11.20125799v1)

			Universidad del Rosario, Bogotá, Colombia	Bogotá, Colombia Instituto Nacional de Salud, Bogotá, Colombia Icahn School of Medicine at Mount Sinai, New York, USA	
**Colombia/GUR-0215/2020**	EPI_ISL_447743	2020-03-28	Grupo de Investigaciones Microbiológicas-UR (GIMUR), Departamento de Biologia, Facultad de Ciencias Naturales, Universidad del Rosario, Bogotá, Colombia	Grupo de Investigaciones Microbiológicas-UR (GIMUR), Departamento de Biologia, Facultad de Ciencias Naturales, Universidad del Rosario, Bogotá, Colombia Instituto Nacional de Salud, Bogotá, Colombia Icahn School of Medicine at Mount Sinai, New York, USA	Juan David Ramirez et al (https://www.medrxiv.org/content/10.1101/2020.06.11.20125799v1)
**Colombia/GUR-0454/2020**	EPI_ISL_447744	2020-03-28	Grupo de Investigaciones Microbiológicas-UR (GIMUR), Departamento de Biologia, Facultad de Ciencias Naturales, Universidad del Rosario, Bogotá, Colombia	Grupo de Investigaciones Microbiológicas-UR (GIMUR), Departamento de Biologia, Facultad de Ciencias Naturales, Universidad del Rosario, Bogotá, Colombia Instituto Nacional de Salud, Bogotá, Colombia Icahn School of Medicine at Mount Sinai, New York, USA	Juan David Ramirez et al (https://www.medrxiv.org/content/10.1101/2020.06.11.20125799v1)
**Colombia/GUV-92155/2020**	EPI_ISL_447762	2020-03-31	Instituto Nacional de Salud, Bogotá, Colombia	Grupo de Investigaciones Microbiológicas-UR (GIMUR), Departamento de Biologia, Facultad de Ciencias Naturales, Universidad del Rosario, Bogotá, Colombia Instituto Nacional de Salud, Bogotá, Colombia Icahn School of Medicine at Mount Sinai, New York, USA	Juan David Ramirez et al (https://www.medrxiv.org/content/10.1101/2020.06.11.20125799v1)

**Colombia/GUV-92218/2020**	EPI_ISL_447765	2020-03-31	Instituto Nacional de Salud, Bogotá, Colombia	Grupo de Investigaciones Microbiológicas-UR (GIMUR), Departamento de Biologia, Facultad de Ciencias Naturales, Universidad del Rosario, Bogotá, Colombia Instituto Nacional de Salud, Bogotá, Colombia Icahn School of Medicine at Mount Sinai, New York, USA	Juan David Ramirez et al (https://www.medrxiv.org/content/10.1101/2020.06.11.20125799v1)
**Colombia/GVI-92161/2020**	EPI_ISL_447763	2020-03-31	Instituto Nacional de Salud, Bogotá, Colombia	Grupo de Investigaciones Microbiológicas-UR (GIMUR), Departamento de Biologia, Facultad de Ciencias Naturales, Universidad del Rosario, Bogotá, Colombia Instituto Nacional de Salud, Bogotá, Colombia Icahn School of Medicine at Mount Sinai, New York, USA	Juan David Ramirez et al (https://www.medrxiv.org/content/10.1101/2020.06.11.20125799v1)
**Colombia/GVI-93301/2020**	EPI_ISL_447769	2020-04-01	Instituto Nacional de Salud, Bogotá, Colombia	Grupo de Investigaciones Microbiológicas-UR (GIMUR), Departamento de Biologia, Facultad de Ciencias Naturales, Universidad del Rosario, Bogotá, Colombia Instituto Nacional de Salud, Bogotá, Colombia Icahn School of Medicine at Mount Sinai, New York, USA	Juan David Ramirez et al (https://www.medrxiv.org/content/10.1101/2020.06.11.20125799v1)
**Colombia/GVI-93450/2020**	EPI_ISL_447777	2020-04-01	Instituto Nacional de Salud, Bogotá, Colombia	Grupo de Investigaciones Microbiológicas-UR (GIMUR), Departamento de Biologia, Facultad de Ciencias Naturales,	Juan David Ramirez et al (https://www.medrxiv.org/content/10.1101/2020.06.11.20125799v1)

				Universidad del Rosario, Bogotá, Colombia Instituto Nacional de Salud, Bogotá, Colombia Icahn School of Medicine at Mount Sinai, New York, USA	
**Colombia/GVI-93457/2020**	EPI_ISL_447778	2020-04-01	Instituto Nacional de Salud, Bogotá, Colombia	Grupo de Investigaciones Microbiológicas-UR (GIMUR), Departamento de Biologia, Facultad de Ciencias Naturales, Universidad del Rosario, Bogotá, Colombia Instituto Nacional de Salud, Bogotá, Colombia Icahn School of Medicine at Mount Sinai, New York, USA	Juan David Ramirez et al (https://www.medrxiv.org/content/10.1101/2020.06.11.20125799v1)
**Colombia/GVI-97718/2020**	EPI_ISL_447800	2020-04-04	Instituto Nacional de Salud, Bogotá, Colombia	Grupo de Investigaciones Microbiológicas-UR (GIMUR), Departamento de Biologia, Facultad de Ciencias Naturales, Universidad del Rosario, Bogotá, Colombia Instituto Nacional de Salud, Bogotá, Colombia Icahn School of Medicine at Mount Sinai, New York, USA	Juan David Ramirez et al (https://www.medrxiv.org/content/10.1101/2020.06.11.20125799v1)
**Colombia/GVI-97795/2020**	**EPI_ISL_447805**	**2020-04-04**	**Instituto Nacional de Salud, Bogotá, Colombia**	**Grupo de Investigaciones Microbiológicas-UR (GIMUR), Departamento de Biologia, Facultad de Ciencias Naturales, Universidad del Rosario, Bogotá, Colombia Instituto Nacional de Salud, Bogotá, Colombia Icahn School of**	**Juan David Ramirez et al (https://www.medrxiv.org/content/10.1101/2020.06.11.20125799v1)**

				**Medicine at Mount Sinai, New York, USA**	
**Colombia/GVI-97855/2020**	EPI_ISL_447806	2020-04-04	Instituto Nacional de Salud, Bogotá, Colombia	Grupo de Investigaciones Microbiológicas-UR (GIMUR), Departamento de Biologia, Facultad de Ciencias Naturales, Universidad del Rosario, Bogotá, Colombia Instituto Nacional de Salud, Bogotá, Colombia Icahn School of Medicine at Mount Sinai, New York, USA	Juan David Ramirez et al (https://www.medrxiv.org/content/10.1101/2020.06.11.20125799v1)
**Colombia/GVI-97875/2020**	EPI_ISL_447807	2020-04-04	Instituto Nacional de Salud, Bogotá, Colombia	Grupo de Investigaciones Microbiológicas-UR (GIMUR), Departamento de Biologia, Facultad de Ciencias Naturales, Universidad del Rosario, Bogotá, Colombia Instituto Nacional de Salud, Bogotá, Colombia Icahn School of Medicine at Mount Sinai, New York, USA	Juan David Ramirez et al (https://www.medrxiv.org/content/10.1101/2020.06.11.20125799v1)
**Colombia/GVI-97921/2020**	EPI_ISL_447808	2020-04-04	Instituto Nacional de Salud, Bogotá, Colombia	Grupo de Investigaciones Microbiológicas-UR (GIMUR), Departamento de Biologia, Facultad de Ciencias Naturales, Universidad del Rosario, Bogotá, Colombia Instituto Nacional de Salud, Bogotá, Colombia Icahn School of Medicine at Mount Sinai, New York, USA	Juan David Ramirez et al (https://www.medrxiv.org/content/10.1101/2020.06.11.20125799v1)
**Colombia/GVI-98087/2020**	EPI_ISL_447812	2020-04-05	Instituto Nacional de Salud, Bogotá, Colombia	Grupo de Investigaciones Microbiológicas-UR (GIMUR), Departamento de	Juan David Ramirez et al (https://www.medrxiv.org

				Biologia, Facultad de Ciencias Naturales, Universidad del Rosario, Bogotá, Colombia Instituto Nacional de Salud, Bogotá, Colombia Icahn School of Medicine at Mount Sinai, New York, USA	/content/10.1101/2020.06.11.20125799v1)
**Colombia/GVI-98290/2020**	EPI_ISL_447813	2020-04-05	Instituto Nacional de Salud, Bogotá, Colombia	Grupo de Investigaciones Microbiológicas-UR (GIMUR), Departamento de Biologia, Facultad de Ciencias Naturales, Universidad del Rosario, Bogotá, Colombia Instituto Nacional de Salud, Bogotá, Colombia Icahn School of Medicine at Mount Sinai, New York, USA	Juan David Ramirez et al (https://www.medrxiv.org/content/10.1101/2020.06.11.20125799v1)
**Colombia/INS-101935/2020**	EPI_ISL_456153	2020-04-22	Instituto Nacional de Salud - Unidad de Secuenciación y Análisis Genómico	Instituto Nacional de Salud, Universidad Cooperativa de Colombia, Instituto Alexander von Humboldt, Imperial College-London, London School of Hygiene & Tropical Medicine	Katherine Laiton-Donato et al A (https://dx.doi.org/10.1101/2020.06.02.20120782)
**Colombia/INS-103039/2020**	EPI_ISL_456144	2020-04-23	Instituto Nacional de Salud - Unidad de Secuenciación y Análisis Genómico	Instituto Nacional de Salud, Universidad Cooperativa de Colombia, Instituto Alexander von Humboldt, Imperial College-London, London School of Hygiene & Tropical Medicine	Katherine Laiton-Donato et al A (https://dx.doi.org/10.1101/2020.06.02.20120782)
**Colombia/INS-79890/2020**	EPI_ISL_456148	2020-03-16	Instituto Nacional de Salud - Unidad de Secuenciación y Análisis Genómico	Instituto Nacional de Salud, Universidad Cooperativa de Colombia, Instituto Alexander von Humboldt, Imperial College-London,	Katherine Laiton-Donato et al A (https://dx.doi.org/10.1101/2020.06.02.20120782)

				London School of Hygiene & Tropical Medicine	
**Colombia/INS-81035/2020**	EPI_ISL_456125	2020-03-20	Instituto Nacional de Salud - Unidad de Secuenciación y Análisis Genómico	Instituto Nacional de Salud, Universidad Cooperativa de Colombia, Instituto Alexander von Humboldt, Imperial College-London, London School of Hygiene & Tropical Medicine	Katherine Laiton-Donato et al A (https://dx.doi.org/10.1101/2020.06.02.20120782)
**Colombia/INS-79190/2020**	EPI_ISL_456120	2020-03-12	Instituto Nacional de Salud - Unidad de Secuenciación y Análisis Genómico	Instituto Nacional de Salud, Universidad Cooperativa de Colombia, Instituto Alexander von Humboldt, Imperial College-London, London School of Hygiene & Tropical Medicine	Katherine Laiton-Donato et al A (https://dx.doi.org/10.1101/2020.06.02.20120782)
**Colombia/GUR-0458/2020**	EPI_ISL_447745	2020-03-14	Grupo de Investigaciones Microbiológicas-UR (GIMUR), Departamento de Biologia, Facultad de Ciencias Naturales, Universidad del Rosario, Bogotá, Colombia	Grupo de Investigaciones Microbiológicas-UR (GIMUR), Departamento de Biologia, Facultad de Ciencias Naturales, Universidad del Rosario, Bogotá, Colombia Instituto Nacional de Salud, Bogotá, Colombia Icahn School of Medicine at Mount Sinai, New York, USA	Katherine Laiton-Donato et al A (https://dx.doi.org/10.1101/2020.06.02.20120782)
**Colombia/GUR-0459/2020**	EPI_ISL_447746	**2020-03-16**	Grupo de Investigaciones Microbiológicas-UR (GIMUR), Departamento de Biologia, Facultad de Ciencias Naturales, Universidad del Rosario, Bogotá, Colombia	Grupo de Investigaciones Microbiológicas-UR (GIMUR), Departamento de Biologia, Facultad de Ciencias Naturales, Universidad del Rosario, Bogotá, Colombia Instituto Nacional de Salud, Bogotá, Colombia Icahn School of Medicine at Mount Sinai, New York, USA	**Katherine Laiton-Donato et al A (https://dx.doi.org/10.1101/2020.06.02.20120782)**

**Colombia/GUR-1215/2020**	EPI_ISL_447754	2020-03-14	Grupo de Investigaciones Microbiológicas-UR (GIMUR), Departamento de Biologia, Facultad de Ciencias Naturales, Universidad del Rosario, Bogotá, Colombia	Grupo de Investigaciones Microbiológicas-UR (GIMUR), Departamento de Biologia, Facultad de Ciencias Naturales, Universidad del Rosario, Bogotá, Colombia Instituto Nacional de Salud, Bogotá, Colombia Icahn School of Medicine at Mount Sinai, New York, USA	Katherine Laiton-Donato et al A (https://dx.doi.org/10.1101/2020.06.02.20120782)
**Colombia/GUV-92034/2020**	EPI_ISL_447755	2020-03-14	Instituto Nacional de Salud, Bogotá, Colombia	Grupo de Investigaciones Microbiológicas-UR (GIMUR), Departamento de Biologia, Facultad de Ciencias Naturales, Universidad del Rosario, Bogotá, Colombia Instituto Nacional de Salud, Bogotá, Colombia Icahn School of Medicine at Mount Sinai, New York, USA	Katherine Laiton-Donato et al A (https://dx.doi.org/10.1101/2020.06.02.20120782)
**Colombia/GUV-92061/2020**	EPI_ISL_447759	2020-03-16	Instituto Nacional de Salud, Bogotá, Colombia	Grupo de Investigaciones Microbiológicas-UR (GIMUR), Departamento de Biologia, Facultad de Ciencias Naturales, Universidad del Rosario, Bogotá, Colombia Instituto Nacional de Salud, Bogotá, Colombia Icahn School of Medicine at Mount Sinai, New York, USA	Katherine Laiton-Donato et al A (https://dx.doi.org/10.1101/2020.06.02.20120782)
**Colombia/GUV-92087/2020**	EPI_ISL_447760	2020-04-06	Instituto Nacional de Salud, Bogotá, Colombia	Grupo de Investigaciones Microbiológicas-UR (GIMUR), Departamento de Biologia, Facultad de Ciencias Naturales,	Juan David Ramirez et al (https://www.medrxiv.org/content/10.1101/2020.06.11.20125799v1)

				Universidad del Rosario, Bogotá, Colombia Instituto Nacional de Salud, Bogotá, Colombia Icahn School of Medicine at Mount Sinai, New York, USA	
**Colombia/GVI-93473/2020**	EPI_ISL_447781	2020-03-31	Instituto Nacional de Salud, Bogotá, Colombia	Grupo de Investigaciones Microbiológicas-UR (GIMUR), Departamento de Biologia, Facultad de Ciencias Naturales, Universidad del Rosario, Bogotá, Colombia Instituto Nacional de Salud, Bogotá, Colombia Icahn School of Medicine at Mount Sinai, New York, USA	Juan David Ramirez et al (https://www.medrxiv.org/content/10.1101/2020.06.11.20125799v1)
**Colombia/GVI97223/2020**	EPI_ISL_447814	2020-04-07	Instituto Nacional de Salud, Bogotá, Colombia	Grupo de Investigaciones Microbiológicas-UR (GIMUR), Departamento de Biologia, Facultad de Ciencias Naturales, Universidad del Rosario, Bogotá, Colombia Instituto Nacional de Salud, Bogotá, Colombia Icahn School of Medicine at Mount Sinai, New York, USA	Juan David Ramirez et al (https://www.medrxiv.org/content/10.1101/2020.06.11.20125799v1)
**Colombia/GVI97517/2020**	EPI_ISL_447816	2020-03-30	Instituto Nacional de Salud, Bogotá, Colombia	Grupo de Investigaciones Microbiológicas-UR (GIMUR), Departamento de Biologia, Facultad de Ciencias Naturales, Universidad del Rosario, Bogotá, Colombia Instituto Nacional de Salud, Bogotá, Colombia Icahn School of	Juan David Ramirez et al (https://www.medrxiv.org/content/10.1101/2020.06.11.20125799v1)

				Medicine at Mount Sinai, New York, USA	
**Colombia/INS-79743/2020**	EPI_ISL_456145	2020-03-16	Instituto Nacional de Salud - Unidad de Secuenciación y Análisis Genómico	Instituto Nacional de Salud, Universidad Cooperativa de Colombia, Instituto Alexander von Humboldt, Imperial College-London, London School of Hygiene & Tropical Medicine	Katherine Laiton-Donato et al A (https://dx.doi.org/10.1101/2020.06.02.20120782)
**Colombia/INS-79943/2020**	**EPI_ISL_456138**	2020-03-14	**Instituto Nacional de Salud - Unidad de Secuenciación y Análisis Genómico**	**Instituto Nacional de Salud, Universidad Cooperativa de Colombia, Instituto Alexander von Humboldt, Imperial College-London, London School of Hygiene & Tropical Medicine**	Katherine Laiton-Donato et al A (https://dx.doi.org/10.1101/2020.06.02.20120782)
**Colombia/INS-79965/2020**	EPI_ISL_456150	2020-03-14	Instituto Nacional de Salud - Unidad de Secuenciación y Análisis Genómico	Instituto Nacional de Salud, Universidad Cooperativa de Colombia, Instituto Alexander von Humboldt, Imperial College-London, London School of Hygiene & Tropical Medicine	Katherine Laiton-Donato et al A (https://dx.doi.org/10.1101/2020.06.02.20120782)
**Colombia/INS-80245/2020**	EPI_ISL_456155	2020-03-17	Instituto Nacional de Salud - Unidad de Secuenciación y Análisis Genómico	Instituto Nacional de Salud, Universidad Cooperativa de Colombia, Instituto Alexander von Humboldt, Imperial College-London, London School of Hygiene & Tropical Medicine	Katherine Laiton-Donato et al A (https://dx.doi.org/10.1101/2020.06.02.20120782)
**Colombia/INS-80656/2020**	EPI_ISL_456126	2020-03-22	Instituto Nacional de Salud - Unidad de Secuenciación y Análisis Genómico	Instituto Nacional de Salud, Universidad Cooperativa de Colombia, Instituto Alexander von Humboldt, Imperial College-London, London School of Hygiene & Tropical Medicine	Katherine Laiton-Donato et al A (https://dx.doi.org/10.1101/2020.06.02.20120782)

**Colombia/INS-82457/2020**	EPI_ISL_456146	2020-03-20	Instituto Nacional de Salud - Unidad de Secuenciación y Análisis Genómico	Instituto Nacional de Salud, Universidad Cooperativa de Colombia, Instituto Alexander von Humboldt, Imperial College-London, London School of Hygiene & Tropical Medicine	Katherine Laiton-Donato et al A (https://dx.doi.org/10.1101/2020.06.02.20120782)
**Colombia/INS-84861/2020**	EPI_ISL_456116	2020-03-31	Instituto Nacional de Salud - Unidad de Secuenciación y Análisis Genómico	Instituto Nacional de Salud, Universidad Cooperativa de Colombia, Instituto Alexander von Humboldt, Imperial College-London, London School of Hygiene & Tropical Medicine	Juan David Ramirez et al (https://www.medrxiv.org/content/10.1101/2020.06.11.20125799v1)
**Colombia/GVI-93537/2020**	EPI_ISL_447789	2020-04-03	Instituto Nacional de Salud, Bogotá, Colombia	Grupo de Investigaciones Microbiológicas-UR (GIMUR), Departamento de Biologia, Facultad de Ciencias Naturales, Universidad del Rosario, Bogotá, Colombia Instituto Nacional de Salud, Bogotá, Colombia Icahn School of Medicine at Mount Sinai, New York, USA	Juan David Ramirez et al (https://www.medrxiv.org/content/10.1101/2020.06.11.20125799v1)
**Colombia/Bogota78390/2020**	EPI_ISL_418262	2020-03-06	Instituto Nacional de Salud	Instituto Nacional de Salud Universidad Cooperativa de Colombia Instituto Alexander von Humboldt Imperial College-London London School of Hygiene & Tropical Medicine	Marcela Mercado-Reyes et al (https://dx.doi.org/10.1101/2020.06.02.20120782)
**Colombia/GUR-0627/2020**	EPI_ISL_447749	**2020-04-04**	Grupo de Investigaciones Microbiológicas-UR (GIMUR), Departamento de Biologia, Facultad de Ciencias Naturales,	Grupo de Investigaciones Microbiológicas-UR (GIMUR), Departamento de Biologia, Facultad de Ciencias Naturales, Universidad del Rosario,	**Juan David Ramirez et al (https://www.medrxiv.org/content/10.1101/2020.06.11.20125799v1)**

			Universidad del Rosario, Bogotá, Colombia	Bogotá, Colombia Instituto Nacional de Salud, Bogotá, Colombia Icahn School of Medicine at Mount Sinai, New York, USA	
**Colombia/GVI-97210/2020**	EPI_ISL_447794	2020-03-18	Instituto Nacional de Salud, Bogotá, Colombia	Grupo de Investigaciones Microbiológicas-UR (GIMUR), Departamento de Biologia, Facultad de Ciencias Naturales, Universidad del Rosario, Bogotá, Colombia Instituto Nacional de Salud, Bogotá, Colombia Icahn School of Medicine at Mount Sinai, New York, USA	Katherine Laiton-Donato et al A (https://dx.doi.org/10.1101/2020.06.02.20120782)
**Colombia/GVI-97321/2020**	**EPI_ISL_447796**	2020-04-01	**Instituto Nacional de Salud, Bogotá, Colombia**	**Grupo de Investigaciones Microbiológicas-UR (GIMUR), Departamento de Biologia, Facultad de Ciencias Naturales, Universidad del Rosario, Bogotá, Colombia Instituto Nacional de Salud, Bogotá, Colombia Icahn School of Medicine at Mount Sinai, New York, USA**	Juan David Ramirez et al (https://www.medrxiv.org/content/10.1101/2020.06.11.20125799v1)
**Colombia/GVI-97417/2020**	EPI_ISL_447798	2020-03-29	Instituto Nacional de Salud, Bogotá, Colombia	Grupo de Investigaciones Microbiológicas-UR (GIMUR), Departamento de Biologia, Facultad de Ciencias Naturales, Universidad del Rosario, Bogotá, Colombia Instituto Nacional de Salud, Bogotá, Colombia Icahn School of Medicine at Mount Sinai, New York, USA	Juan David Ramirez et al (https://www.medrxiv.org/content/10.1101/2020.06.11.20125799v1)

**Colombia/GVI-97769/2020**	EPI_ISL_447802	2020-03-15	Instituto Nacional de Salud, Bogotá, Colombia	Grupo de Investigaciones Microbiológicas-UR (GIMUR), Departamento de Biologia, Facultad de Ciencias Naturales, Universidad del Rosario, Bogotá, Colombia Instituto Nacional de Salud, Bogotá, Colombia Icahn School of Medicine at Mount Sinai, New York, USA	Katherine Laiton-Donato et al A (https://dx.doi.org/10.1101/2020.06.02.20120782)
**Colombia/GVI-97782/2020**	**EPI_ISL_447803**	2020-04-04	**Instituto Nacional de Salud, Bogotá, Colombia**	**Grupo de Investigaciones Microbiológicas-UR (GIMUR), Departamento de Biologia, Facultad de Ciencias Naturales, Universidad del Rosario, Bogotá, Colombia Instituto Nacional de Salud, Bogotá, Colombia Icahn School of Medicine at Mount Sinai, New York, USA**	Juan David Ramirez et al (https://www.medrxiv.org/content/10.1101/2020.06.11.20125799v1)
**Colombia/GVI-97783/2020**	EPI_ISL_447804	2020-03-29	Instituto Nacional de Salud, Bogotá, Colombia	Grupo de Investigaciones Microbiológicas-UR (GIMUR), Departamento de Biologia, Facultad de Ciencias Naturales, Universidad del Rosario, Bogotá, Colombia Instituto Nacional de Salud, Bogotá, Colombia Icahn School of Medicine at Mount Sinai, New York, USA	Juan David Ramirez et al (https://www.medrxiv.org/content/10.1101/2020.06.11.20125799v1)
**Colombia/INS-103303/2020**	EPI_ISL_456154	**2020-04-04**	Instituto Nacional de Salud - Unidad de Secuenciación y Análisis Genómico	Instituto Nacional de Salud, Universidad Cooperativa de Colombia, Instituto Alexander von Humboldt, Imperial College-London,	**Juan David Ramirez et al (https://www.medrxiv.org/content/10.1101/2020.06.11.20125799v1)**

				London School of Hygiene & Tropical Medicine	
**Colombia/INS-80123/2020**	EPI_ISL_456149	2020-04-03	Instituto Nacional de Salud - Unidad de Secuenciación y Análisis Genómico	Instituto Nacional de Salud, Universidad Cooperativa de Colombia, Instituto Alexander von Humboldt, Imperial College-London, London School of Hygiene & Tropical Medicine	Juan David Ramirez et al (https://www.medrxiv.org/content/10.1101/2020.06.11.20125799v1)
**Colombia/INS-81251/2020**	EPI_ISL_456139	2020-04-26	Instituto Nacional de Salud - Unidad de Secuenciación y Análisis Genómico	Instituto Nacional de Salud, Universidad Cooperativa de Colombia, Instituto Alexander von Humboldt, Imperial College-London, London School of Hygiene & Tropical Medicine	Katherine Laiton-Donato et al A (https://dx.doi.org/10.1101/2020.06.02.20120782)
**Colombia/Cali-01/2020**	EPI_ISL_445085	2020-04-01	unknown	Virology Unit	Juan David Ramirez et al (https://www.medrxiv.org/content/10.1101/2020.06.11.20125799v1)
**Colombia/Cali-01-2/2020**	EPI_ISL_445219	2020-03-10	Universidad del Valle, Laboratorio de Microbiologia, VIREM	Universidad del Valle, Universidad Nacional de Colombia-Sede Palmira, International Center for Tropical Agriculture	Katherine Laiton-Donato et al A (https://dx.doi.org/10.1101/2020.06.02.20120782)
**Colombia/GUR-0084/2020**	EPI_ISL_447735	**2020-04-04**	Grupo de Investigaciones Microbiológicas-UR (GIMUR), Departamento de Biologia, Facultad de Ciencias Naturales, Universidad del Rosario, Bogotá, Colombia	Grupo de Investigaciones Microbiológicas-UR (GIMUR), Departamento de Biologia, Facultad de Ciencias Naturales, Universidad del Rosario, Bogotá, Colombia Instituto Nacional de Salud, Bogotá, Colombia Icahn School of Medicine at Mount Sinai, New York, USA	**Juan David Ramirez et al (https://www.medrxiv.org/content/10.1101/2020.06.11.20125799v1)**
**Colombia/GUR-0787/2020**	EPI_ISL_447750	2020-04-02	Grupo de Investigaciones Microbiológicas-UR	Grupo de Investigaciones Microbiológicas-UR	Lopez et al

			(GIMUR), Departamento de Biologia, Facultad de Ciencias Naturales, Universidad del Rosario, Bogotá, Colombia	(GIMUR), Departamento de Biologia, Facultad de Ciencias Naturales, Universidad del Rosario, Bogotá, Colombia Instituto Nacional de Salud, Bogotá, Colombia Icahn School of Medicine at Mount Sinai, New York, USA	
**Colombia/GUV-92250/2020**	EPI_ISL_447766	2020-04-01	Instituto Nacional de Salud, Bogotá, Colombia	Grupo de Investigaciones Microbiológicas-UR (GIMUR), Departamento de Biologia, Facultad de Ciencias Naturales, Universidad del Rosario, Bogotá, Colombia Instituto Nacional de Salud, Bogotá, Colombia Icahn School of Medicine at Mount Sinai, New York, USA	Katherine Laiton-Donato et al A (https://dx.doi.org/10.1101/2020.06.02.20120782)
**Colombia/GUV-92256/2020**	EPI_ISL_447768	2020-04-04	Instituto Nacional de Salud, Bogotá, Colombia	Grupo de Investigaciones Microbiológicas-UR (GIMUR), Departamento de Biologia, Facultad de Ciencias Naturales, Universidad del Rosario, Bogotá, Colombia Instituto Nacional de Salud, Bogotá, Colombia Icahn School of Medicine at Mount Sinai, New York, USA	Juan David Ramirez et al (https://www.medrxiv.org/content/10.1101/2020.06.11.20125799v1)
**Colombia/GVI-93352/2020**	EPI_ISL_447771	2020-04-03	Instituto Nacional de Salud, Bogotá, Colombia	Grupo de Investigaciones Microbiológicas-UR (GIMUR), Departamento de Biologia, Facultad de Ciencias Naturales, Universidad del Rosario, Bogotá, Colombia Instituto	Juan David Ramirez et al (https://www.medrxiv.org/content/10.1101/2020.06.11.20125799v1)

				Nacional de Salud, Bogotá, Colombia Icahn School of Medicine at Mount Sinai, New York, USA	
**Colombia/GVI-97203/2020**	EPI_ISL_447793	2020-03-26	Instituto Nacional de Salud, Bogotá, Colombia	Grupo de Investigaciones Microbiológicas-UR (GIMUR), Departamento de Biologia, Facultad de Ciencias Naturales, Universidad del Rosario, Bogotá, Colombia Instituto Nacional de Salud, Bogotá, Colombia Icahn School of Medicine at Mount Sinai, New York, USA	Juan David Ramirez et al (https://www.medrxiv.org/content/10.1101/2020.06.11.20125799v1)
**Colombia/GVI-97212/2020**	EPI_ISL_447795	2020-04-03	Instituto Nacional de Salud, Bogotá, Colombia	Grupo de Investigaciones Microbiológicas-UR (GIMUR), Departamento de Biologia, Facultad de Ciencias Naturales, Universidad del Rosario, Bogotá, Colombia Instituto Nacional de Salud, Bogotá, Colombia Icahn School of Medicine at Mount Sinai, New York, USA	Juan David Ramirez et al (https://www.medrxiv.org/content/10.1101/2020.06.11.20125799v1)
**Colombia/GVI-98006/2020**	**EPI_ISL_447809**	**2020-03-30**	**Instituto Nacional de Salud, Bogotá, Colombia**	**Grupo de Investigaciones Microbiológicas-UR (GIMUR), Departamento de Biologia, Facultad de Ciencias Naturales, Universidad del Rosario, Bogotá, Colombia Instituto Nacional de Salud, Bogotá, Colombia Icahn School of Medicine at Mount Sinai, New York, USA**	**Juan David Ramirez et al (https://www.medrxiv.org/content/10.1101/2020.06.11.20125799v1)**

**Colombia/GVI-98011/2020**	EPI_ISL_447810	2020-04-01	Instituto Nacional de Salud, Bogotá, Colombia	Grupo de Investigaciones Microbiológicas-UR (GIMUR), Departamento de Biologia, Facultad de Ciencias Naturales, Universidad del Rosario, Bogotá, Colombia Instituto Nacional de Salud, Bogotá, Colombia Icahn School of Medicine at Mount Sinai, New York, USA	Juan David Ramirez et al (https://www.medrxiv.org/content/10.1101/2020.06.11.20125799v1)
**Colombia/INS-82320/2020**	EPI_ISL_456151	2020-04-01	Instituto Nacional de Salud - Unidad de Secuenciación y Análisis Genómico	Instituto Nacional de Salud, Universidad Cooperativa de Colombia, Instituto Alexander von Humboldt, Imperial College-London, London School of Hygiene & Tropical Medicine	Juan David Ramirez et al (https://www.medrxiv.org/content/10.1101/2020.06.11.20125799v1)
**Ecuador/USFQ-096/2020**	EPI_ISL_477015	2020-06-17	Institute of Microbiology, Universidad San Francisco de Quito	Institute of Microbiology, Universidad San Francisco de Quito	Sully Márquez et al
**Ecuador/USFQ-097/2020**	EPI_ISL_477016	2020-06-17	Institute of Microbiology, Universidad San Francisco de Quito	Institute of Microbiology, Universidad San Francisco de Quito	Juan José Guadalupe et al
**Ecuador/USFQ-004/2020**	EPI_ISL_477014	2020-03-30	Institute of Microbiology, Universidad San Francisco de Quito	Institute of Microbiology, Universidad San Francisco de Quito	Belen Prado-Vivar et al A
**Ecuador/USFQ-020/2020**	EPI_ISL_471267	2020-04-17	Hospital IESS Babahoyo	Institute of Microbiology, Universidad San Francisco de Quito	Sully Márquez et al
**Ecuador/USFQ-707/2020**	EPI_ISL_471269	2020-06-04	Hospital Oncológico Solca Núcleo de Quito	Institute of Microbiology, Universidad San Francisco de Quito	Sully Márquez et al
**Ecuador/USFQ-106/2020**	EPI_ISL_471271	2020-05-19	Hospital Oncológico Solca Núcleo de Quito	Institute of Microbiology, Universidad San Francisco de Quito	Sully Márquez et al

**Ecuador/USFQ-112/2020**	EPI_ISL_471270	2020-05-19	Hospital Oncológico Solca Núcleo de Quito	Institute of Microbiology, Universidad San Francisco de Quito	Sully Márquez et al
**Ecuador/USFQ-045/2020**	EPI_ISL_471268	2020-04-17	Hospital IESS Babahoyo	Institute of Microbiology, Universidad San Francisco de Quito	Belén Prado-Vivar et al
**Peru/010/2020**	EPI_ISL_415787	2020-03-10	Laboratorio de Referencia Nacional de Virus Respiratorio. Instituto Nacional de Salud. Peru	Laboratorio de Referencia Nacional de Biotecnologia y Biologia Molecular.Instituto Nacional de Salud.Peru	Carlos Padilla Rojas et al (https://dx.doi.org/10.1101/2020.05.23.20111443)
**Uruguay/UY-NYUMC876/2020**	EPI_ISL_457972	2020-04-21	Laboratorio de Biología Molecular Asociación Española Primera en Salud	Departments of Pathology and Medicine, New York University School of Medicine	Maria Victoria Elizondo et al
**Uruguay/UY-NYUMC877/2020**	EPI_ISL_457973	2020-04-22	Laboratorio de Biología Molecular Asociación Española Primera en Salud	Departments of Pathology and Medicine, New York University School of Medicine	Maria Victoria Elizondo et al
**Uruguay/UY-NYUMC873/2020**	EPI_ISL_457969	2020-04-14	Laboratorio de Biología Molecular Asociación Española Primera en Salud	Departments of Pathology and Medicine, New York University School of Medicine	Maria Victoria Elizondo et al
**Uruguay/UY-NYUMC861/2020**	EPI_ISL_457957	2020-03-25	Laboratorio de Biología Molecular Asociación Española Primera en Salud	Departments of Pathology and Medicine, New York University School of Medicine	Maria Victoria Elizondo et al
**Uruguay/UY-NYUMC867/2020**	EPI_ISL_457963	2020-03-28	Laboratorio de Biología Molecular Asociación Española Primera en Salud	Departments of Pathology and Medicine, New York University School of Medicine	Maria Victoria Elizondo et al
**Uruguay/UY-NYUMC857/2020**	EPI_ISL_457953	2020-03-23	Laboratorio de Biología Molecular Asociación Española Primera en Salud	Departments of Pathology and Medicine, New York University School of Medicine	Maria Victoria Elizondo et al
